# Parents Develop Long‐Term Disgust Habituation, but Only After Beginning to Wean Their Children

**DOI:** 10.1111/sjop.70069

**Published:** 2026-01-06

**Authors:** Yifan Huang, Ivo E. Dalmaijer‐Denning, Joris A. Dalmaijer‐Denning, Thomas Armstrong, Edwin S. Dalmaijer

**Affiliations:** ^1^ School of Psychological Science University of Bristol Bristol UK; ^2^ University Day Nursery University of Bristol Bristol UK; ^3^ Department of Psychology Whitman College Walla Walla Washington USA

**Keywords:** contamination, disgust avoidance, disgust; habituation, parenting

## Abstract

Disgust helps humans avoid potentially pathogenic substances such as bodily effluvia. This reduces illness risks and is difficult to overcome with cognitive strategies or through short‐term habituation (minutes to hours). Whether long‐term habituation (months to years) exists is an unsolved question. While regular professional exposure to disgust elicitors is associated with lower disgust sensitivity and avoidance, this could reflect selection and survivorship bias. We use the natural quasi‐experiment of parenthood: it greatly increases exposure to bodily effluvia, but disgust does not usually inspire individuals to start or stop being a parent. Parents (*N* = 99) and controls (*N* = 50) completed self‐report and behavioral avoidance measures. We used parent‐specific items in disgust‐sensitivity questionnaires, and child‐related stimuli (soiled diapers) in a preferential‐looking task. These included diapers with pre‐weaning (yellow and runny) or post‐weaning feces (adult‐like). While the control group showed the expected behavioral avoidance, parents of weaning or weaned children showed almost no avoidance of stimuli depicting child‐related or general bodily effluvia. These results suggest that parents habituated to disgust induced by feces in diapers, and that this had generalized to other bodily effluvia. Contrary to our expectations, parents of pre‐weaning children showed similar disgust avoidance to the control group, even if they had older children. This could point to an adaptive response to reduce the risk of illness in young infants. After the sensitive milk‐feeding stage, continuous exposure to their children's bodily effluvia inoculates parents to disgust.

## Key Points

1. Parents to young children face daily exposure to bodily effluvia.
2. Presumably as a consequence, parents showed markedly reduced disgust avoidance.
3. This disgust habituation only emerged after their children started weaning.
4. Parental disgust habituation generalised to bodily effluvia that were not associated with children.

## 1 Introduction

The emotion of disgust occurs in response to stimuli that could carry pathogens, seemingly to help individuals avoid the risk of contamination (Angyal 1941; Darwin 1872). It is theorized that disgust evolved under disease threat (Curtis and Biran 2001; Rozin and Fallon 1987; Tybur et al. 2013), and computer simulations support this view (Dalmaijer and Armstrong 2020). However, disgust is not necessarily helpful in many modern situations. For example, disgust inspired by professional exposure to body envelope violations or bodily effluvia is merely unpleasant when hygienic procedures are already observed, and we might thus see it decline with experience (Edgar et al. 2024).

Indeed, working with human cadavers (Rozin 2008) or animal meat (Piazza et al. 2021) is associated with lower self‐reported disgust for specifically those stimuli. In addition, medical professionals show lower disgust sensitivity (Schnapp et al. 2019), lower disengagement from patients due to disgust with their symptoms (Reynolds et al. 2019), and reduced behavioral avoidance of core and gore disgust stimuli (Edgar et al. 2024). Even listening to extreme metal music (with potentially disgust‐eliciting lyrics and cover images) has been associated with lower disgust sensitivity for bodily effluvia and body envelope violations (Wabnegger and Schienle 2025). These findings align with the idea that prolonged exposure to disgust elicitors leads to habituation.

While it seems reasonable to assume humans habituate to disgust elicitors with prolonged exposure, there is reason to be skeptical of this claim. Where most emotions have abstract elicitors and are met with flexible responses, disgust is primarily roused by concrete properties of stimuli (e.g., their sight, smell, or taste) and is met with stereotyped responses (e.g., avoidance and proto‐nausea; Royzman and Sabini 2001). This awkward midway point between drive and fully‐fledged emotion makes disgust “cognitively impenetrable” (Royzman and Sabini 2001). As a consequence, disgust resists cognitive restructuring (Mason and Richardson 2012), extinction and counter‐conditioning (Engelhard et al. 2014), and even basic habituation through exposure with or without monetary incentive (Dalmaijer et al. 2021). In fact, only when stomach rhythms are pharmacologically normalized do individuals begin to show reduced disgust avoidance under repeated incentivized exposure (Nord et al. 2021). In sum, habituation to disgust would be the exception rather than the norm in the existing literature.

An additional problem with studies on long‐term disgust habituation is their focus on individuals who made an active choice to be in their profession. Medical students with lower disgust sensitivity are more likely to choose specialities associated with higher disgust exposure, such as nursing and surgery (Consedine, Yu, Hill, and Windsor 2013; Consedine, Yu, and Windsor 2013; Consedine and Windsor 2014). In addition to self‐selecting into specific professions, individuals with lower disgust sensitivity could be less likely to change occupation. There is thus both selection bias and survivorship bias, which could explain lower and reducing disgust sensitivity in these professions. In other words: maybe individuals do not habituate to disgust, but simply self‐select.

Here, we attempted to avoid selection and survivorship bias by sampling from a group whose members do not typically weigh disgust into their decision to join: parents. Caring for young children requires frequent and long‐term exposure to bodily effluvia. While it has been shown that mothers are less disgusted by feces produced by their own compared to others' infants (Case et al. 2006), there is only preliminary evidence that this “source effect” translates into a generalized reduced disgust sensitivity among mothers compared to childless women (Prokop and Fančovičová 2016; Stefanczyk et al. 2024). With this study, we aimed to investigate whether parents habituate to specific child‐associated stimuli, and whether this generalizes to other bodily effluvia.

Specifically, we leveraged a natural quasi‐experiment in which parents are subjected to qualitatively different types of feces as their children age. After a very brief initial period of producing meconium that is uniquely dark and sticky, infants produce feces characterized by a stereotypically yellow to green coloring and texturing through fat (and potentially casein) curds (Bekkali et al. 2009; Gustin et al. 2018; Talbot 1910). After about 6 months, infants move from a fully milk‐based diet to more solid foods. This process of weaning has a profound impact on the appearance of infant feces, which become more offensive in smell and appearance, and increasingly alike adult stool. If disgust habituation is indeed of highly limited generalization (Rozin 2008), parents would habituate specifically to the stool that their children produce. Parents of milk‐fed infants would thus show reduced avoidance of “milk diapers”, but remain avoidant of diapers with post‐weaning feces. Similarly, parents of children who eat solid foods would show reduced avoidance of “weaning diapers”.

In addition to opening a window into the acquisition of disgust habituation, the natural quasi‐experiment of parenting young children also offers an opportunity to test its longevity. Parents are typically only faced with milk diapers for up to 6 months. If disgust habituation persists, then they should continue to show reduced avoidance of these stimuli. Similarly, parents whose children no longer wear diapers should continue to show reduced avoidance of diaper stimuli. Alternatively, if parents lose their habituation to disgusting diapers, their avoidance of such stimuli should return toward typical levels.

Finally, parental disgust avoidance behavior can be used to gauge the generalization of disgust habituation. Healthcare assistants who work in care homes show no behavioral avoidance of stimuli frequently encountered in their professional capacity, but also fail to avoid unrelated core and gore disgust elicitors (Edgar et al. 2024). This could reflect generalization of disgust habituation to a wider class of stimuli, but could also be a result of aforementioned selection and survivorship biases that make it less likely for individuals with higher disgust sensitivity to be and remain in disgust‐prone professions. Parents, on the other hand, have limited choice in the matter. It seems unlikely they would forego or opt out of parenthood for reasons of disgust sensitivity. Further, while the source effect might help reduce disgust for their own infant's feces (Case et al. 2006), it (by definition) should not extend to other infants' diapers or other displays of bodily effluvia. Hence, generalization of disgust habituation could be directly tested by quantifying the extent to which parents avoid feces or vomit from sources other than children.

In sum, this study focused on behavioral disgust avoidance in parents of children of various ages, in order to test (1) their habituation to diapers containing feces of specific appearances, (2) the longevity of their habituation, and (3) generalization of their habituation.

## 2 Methods

### 2.1 Participants

Two samples were collected as part of this study: parents and a control group of non‐parents. They were recruited through Prolific Academic between 18 March 2024 and 20 August 2024, with permission from the University of Bristol's School of Psychological Science Research Ethics Committee (approval code 17317).

For the purposes of this study, the role of “parent” was self‐defined by participants through screening questions on the Prolific Academic recruitment platform, and confirmed within our study by the question “How many children do you have?”. We also directly asked about disgust exposure by asking how many diapers parent participants changed on an average day.

We did not specifically ask if participants were a biological parent to their children, as we did not think this was important to our research question: our hypothesized mechanism is through exposure to bodily effluvia, which adoptive and biological parents encounter alike. Furthermore, if hormonal effects were different between biological and adoptive parents, those do not necessarily translate to differences in disgust sensitivity (Jones et al. 2018; Stern and Shiramizu 2022). Finally, the number of adoptive parents is low: for example, only 0.7% of children are looked after by a local authority in England, and only 3.6% of those in the care system left through adoption in 2024 (Department for Education 2024).

The parent sample comprised 99 individuals, 26% of whom identified as man, 73% as woman, and 1% as non‐binary. Their ages ranged from 21 to 69, with an average of 37 and a standard deviation of 10 years. 6% of this sample identified as Asian, 20% as Black, 2% as part of an indigenous group, 2% as Pacific Islander, and 69% as White; and 6% reported mixed identities (these were double‐counted). 41% of parents reported having one child, 35% reported 2 children, 14% reported 3, 6% reported 4, 2% reported 5, and 1% reported 6 or more.

Two sub‐groups of parents will be used in this study (see “Data reduction and statistical analysis”). The first comprised 28 parents (25% men) with pre‐weaning children. Their ages ranged from 24 to 55, with an average of 31 and a standard deviation of 6 years. The second sub‐group comprised 71 parents (27% men) with weaned children. Their ages ranged from 21 to 69, with an average of 39 and a standard deviation of 11 years.

The control group comprised 50 individuals, 26% of whom identified as men and 74% as women. Their ages ranged from 18 to 73, with an average of 34 and a standard deviation of 13 years. 28% of this sample identified as Asian, 10% as Black, 2% as part of an indigenous group, 6% as Latine, and 56% as White; and 6% reported mixed identities (these were double‐counted).

Sex and gender differences have been reported in previous studies of self‐reported disgust sensitivity (Tybur et al. 2009), but not in the type of behavioral disgust avoidance task used here (Dalmaijer et al. 2021). Fortunately, the gender composition matched closely between our samples. The samples were also relatively well matched in age and ethnicity.

### 2.2 Procedure

Participants were recruited through Prolific Academic. This recruitment platform pre‐screens its participant pool and allows researchers to only make studies available to subsets. We selected parents on the basis of their country of residence (United Kingdom or United States of America), whether individuals reported having children (“Yes” for parent group and “No” for control group), and the year of birth of their youngest child (oversampling 2024 and 2023 as weaning occurs early, and many parents were likely to already have another weaned child).

From the perspective of potential participants, a study appeared in a list of potential studies. After being redirected from Prolific to the Gorilla experiment platform, participants were provided with an information sheet. They provided informed consent before participation in the study.

Participants first completed a parent questionnaire. This was limited to a single question for the control group (“How many children do you have?”), and participants who answered any number other than “none” were presented with additional questions on their children's ages and diapers (see under “Parent questionnaire” for details). The next element in the study was a preferential looking task, in which participants used a cursor‐guided aperture to explore displays with side‐by‐side stimulus images (see under “Behavioural disgust avoidance” for details). After the task, participants were asked to complete a disgust sensitivity questionnaire (see under “Self‐reported disgust sensitivity” for details), followed by an extension to this questionnaire with items relating to disgust‐inducing situations that parents could face (see under “Self‐reported parenting disgust sensitivity” for details).

At the end of the study, a debrief was provided with (reminders of) information on the study's purposes, procedures, and the researchers' contact information. Participation generally took 20–25 min, and participants were compensated for their time with £3.50.

### 2.3 Parent Questionnaire

Every participant was asked about their age in years, gender, and ethnicity; and then about the number of children they have. Those who answered “none” progressed immediately to the behavioral avoidance task. Those who answered 1 or over were presented with an additional questionnaire.

This parent status questionnaire asked about the age (in months) of their youngest (or only) child, as well as the age (in years) of their oldest (if any). It then asked about the average daily diaper changes, the type of diapers participants used (reusable cloth nappies, single‐use nappies, or a mixture), how disgusting participants found diaper changes for urine‐only and for feces‐containing diapers (on the same scale as used in the Disgust Scale—revised; see next heading), the stage of weaning of their youngest child, and whether any of their children were fully weaned.

Participants could also answer open questions: one that asked to briefly describe any changes they noticed during the weaning process, and another that asked whether participants had any difficulties with diaper changes and how they coped. The purpose of these was not to do any substantial qualitative analysis, but rather to check our assumptions around weaning‐related changes and parental approaches to diaper‐changing.

### 2.4 Self‐Reported Disgust Sensitivity

Disgust sensitivity was quantified using the Disgust Scale—revised (Haidt et al. 1994; Olatunji et al. 2007). This questionnaire contains 27 items, 2 of which are intended as catch questions. In its latest version, participants are asked to respond on a 5‐point scale that is scored 0 through 4: “Strongly disagree (very untrue about me)”, “Mildly disagree (somewhat untrue about me)”, “Neither agree nor disagree”, “Mildly agree (somewhat true about me)”, and “Strongly agree (very true about me)”. The final disgust sensitivity score is the average of 25 disgust‐related items (after reverse‐coding 3 of them).

The scale is sub‐divided into three sub‐scales of core, animal‐reminder, and contamination disgust. However, the conceptual difference between these is relatively subtle; each sub‐scale has lower internal consistency (van Overveld et al. 2011), and they are not necessarily invariant between different countries (Olatunji et al. 2009). While we do not mean to dismiss usage of the sub‐scales for other purposes, we opted for simply computing the total average score to quantify disgust sensitivity.

The score of the revised version of the Disgust Scale was a reliable instrument in different countries within the last two decades: In the United States of America (*α* = 0.87, 0.87, and 0.88; Olatunji et al. 2007); in the Netherlands (Cronbach's *α* = 0.70) and Belgium and the Netherlands (*α* = 0.87) (van Overveld et al. 2011); and in Australia (*α* = 0.80), Brazil (*α* = 0.82), Germany (*α* = 0.75), Italy (*α* = 0.80), Japan (*α* = 0.77), the Netherlands (*α* = 0.68), and Sweden (*α* = 0.78) (Olatunji et al. 2009). It also correlates well with other disgust sensitivity questionnaires (Olatunji et al. 2007; van Overveld et al. 2011) and with behavioral disgust avoidance (Dalmaijer et al. 2021).

### 2.5 Self‐Reported Parenting Disgust Sensitivity

We used an addition to the revised Disgust Scale specific to the parenting domain (Visconti 2013). Items on this questionnaire describe situations related to parenting, for example, being vomited on and changing diapers before dinner. This Parenting Domain Disgust Scale was designed to have the same conceptual coverage as the Disgust Scale—Revised (i.e., the same sub‐scales of core, animal‐reminder, and contamination). While the sub‐scales correlate between the two questionnaires (Visconti 2013), we again elected to simply use the total average score.

While only powered to detect relatively large differences (*N* = 39 parents and *N* = 114 non‐parents), the study that introduced this questionnaire found no difference between parents and non‐parents on disgust sensitivity scores, but it did find that parents scored lower specifically on parenting‐related disgust sensitivity (Visconti 2013). To our knowledge, no further research has been conducted with or on this questionnaire.

### 2.6 Behavioral Disgust Avoidance

To measure disgust avoidance, we employed a web‐based adaptation of a preferential looking task. This type of task shows two images side‐by‐side, and allows participants to view them without restrictions. When one of the images is an elicitor of pathogen disgust, individuals typically look at that stimulus less than at the control stimulus (Armstrong et al. 2014; Dalmaijer et al. 2021), even when accounting for low‐level visual saliency (Dalmaijer et al. 2021). This pattern of sustained avoidance is unique to disgust and does not appear with other highly negatively valenced stimuli such as those associated with threat (Dalmaijer et al. 2021) or suicide (Armstrong et al. 2022).

The variable of interest was dwell time: the total time spent gazing at either stimulus (disgust or neutral). Behavioral disgust avoidance was measured as the difference in dwell time proportions for a disgusting compared to a neutral stimulus. This metric typically shows an early bias towards the disgusting stimulus, but this subsides within 2 s to make place for sustained avoidance over the rest of the trial and subsequent trials (Armstrong et al. 2022; Dalmaijer et al. 2021; Nord et al. 2021). For statistical tests, the dwell time proportions for each stimulus were used separately (they do not add up to 1, as participants also move their cursor to space between and around the images).

**FIGURE 1:**
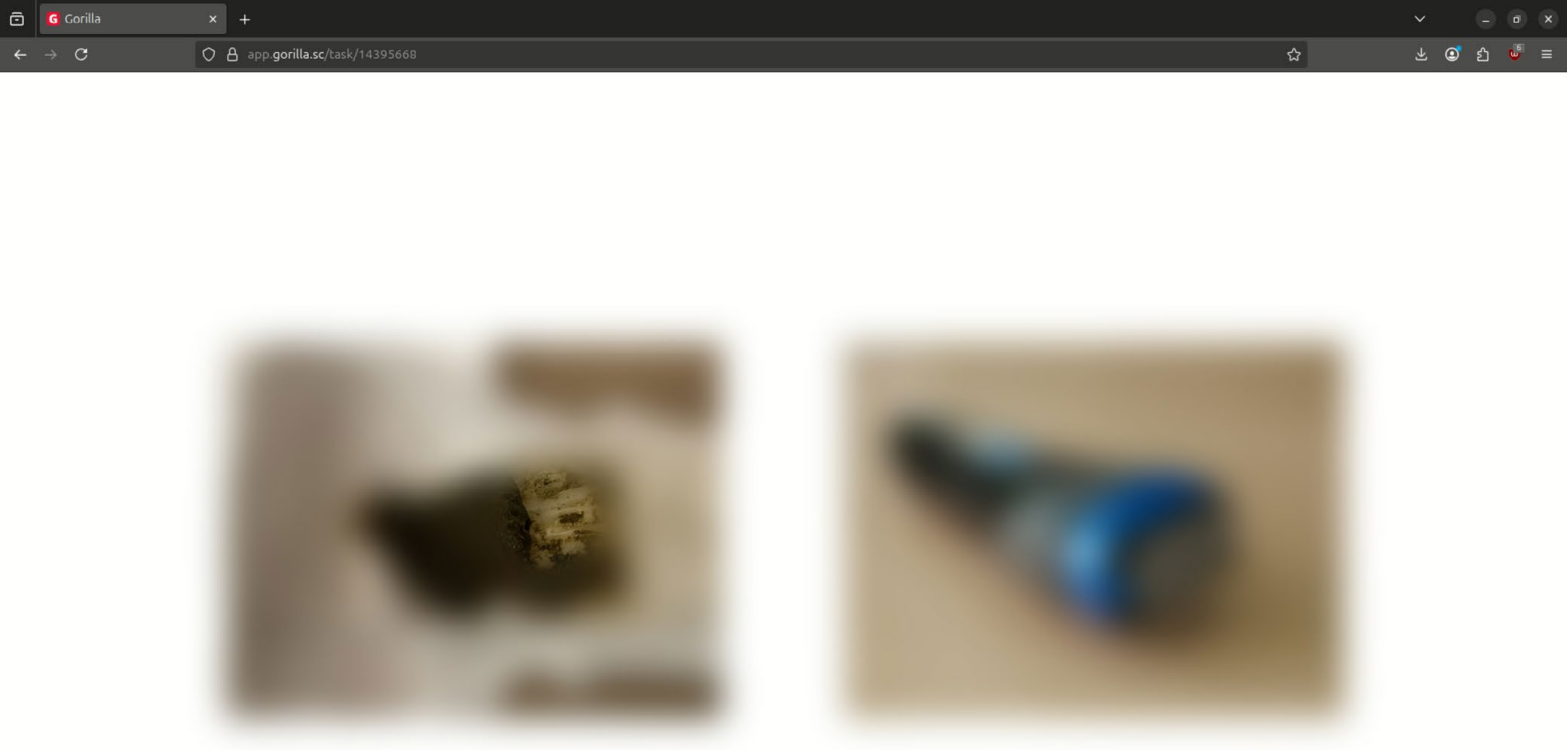
This screenshot shows a single trial in the current study. Two images are shown side‐by‐side: one disgust elicitor (here on the left) and one neutral (here on the right). The stimulus display is blurred, with the exception of an aperture around the cursor (here shown over the left image).

To more easily reach a wider sample, we employed a web‐based preferential looking task in which participants move a cursor‐locked aperture over an otherwise blurred display (Figure 1). This approach has been validated for exactly this use‐case, showing high correlations between cursor and eye dwell time, similar correlations between cursor or eye dwell time and self‐reported disgust, and high internal consistency (Anwyl‐Irvine et al. 2022). The task was implemented using MouseView.js (Anwyl‐Irvine et al. 2022) in the Gorilla experiment builder (Anwyl‐Irvine et al. 2020), which has appropriate timing accuracy for this type of design (Anwyl‐Irvine et al. 2021; Bridges et al. 2020).

We used the same approach as in previous work (Anwyl‐Irvine et al. 2022; Edgar et al. 2024), setting MouseView.js' aperture size to 10% of the screen space and the standard deviation of its Gaussian blur to 20 pixels. Image size depended on participants' distance from their screen and their display settings. In general, the centres of each image were at one‐third and two‐thirds of screen width and half the screen height. Their width was 25% of the screen width, and their height was set to maintain an aspect ratio of 1024 by 768. Stimulus pairs were visible for 10 s in each trial. They were preceded by a marker in the centre of the display, which required participants to click it before the task proceeded. This ensured a central “fixation” at the start of each trial.

Experimental tasks can be less reliable than self‐report measures (Dang et al. 2020), but preferential looking tasks are an exception to this rule. Measuring disgust avoidance in this way has a high reliability: internal consistency estimates range from Spearman‐Brown *ρ* = 0.81 to 0.94 and Cronbach's *α* = 0.78 to 0.91 (Anwyl‐Irvine et al. 2022), and its test–retest reliability is equal to that of self‐reported disgust (Dalmaijer et al. 2021). Measuring behavioral disgust avoidance in this way also has a high validity: it correlates well (*R* = 0.19 to 0.50) with self‐reported disgust for the same stimuli (Anwyl‐Irvine et al. 2022), and reasonably (*R* = 0.28 to 0.34) with self‐reported disgust sensitivity on the revised Disgust Scale (Dalmaijer et al. 2021).

A benefit of measuring behavioral disgust avoidance over self‐reported disgust is that it is less susceptible to demand effects: where self‐report is subject to a very large (Cohen's *d* = 1.25) placebo effect, there is only a small to medium (*d* = 0.44) placebo effect for oculomotor disgust avoidance (Schienle et al. 2016).

### 2.7 Stimuli

For this study, we required a very specific set of stimuli: images of diapers with feces from different stages of development. As these were not readily available, we generated a new stimulus set with photos of diapers with meconium (2), with feces from infants on milk‐only diets (5), and with feces from infants and toddlers throughout the weaning process (13). Their relative numbers reflect the duration of parental exposure to them, as we aimed to sample stimuli across the pre‐weaning and weaning periods.

In addition to parenting‐specific stimuli, we used 5 images of general bodily effluvia. These included 1 image of fecal matter with unidentified white elements lying in grass (used in e.g., Dalmaijer et al. 2021), 2 images of adult feces portraying diarrhea or more solid material in an unkempt toilet (sourced from Haberkamp et al. 2017), and 2 images of vomit on pavement (from Haberkamp et al. 2017) or during emission from an adult's mouth (used in e.g., Anwyl‐Irvine et al. 2022).

Neutral stimuli were sourced from the DIRTI stimulus set (Haberkamp et al. 2017), with the exception of two that were sourced from past research (Anwyl‐Irvine et al. 2022; Dalmaijer et al. 2021). These stimuli portray non‐disgusting scenes or objects that appear visually somewhat similar to avoid gross visual differences, although low‐level visual saliency has a limited effect on dwell times in similar preferential looking tasks (Dalmaijer et al. 2021).

**FIGURE 2:**
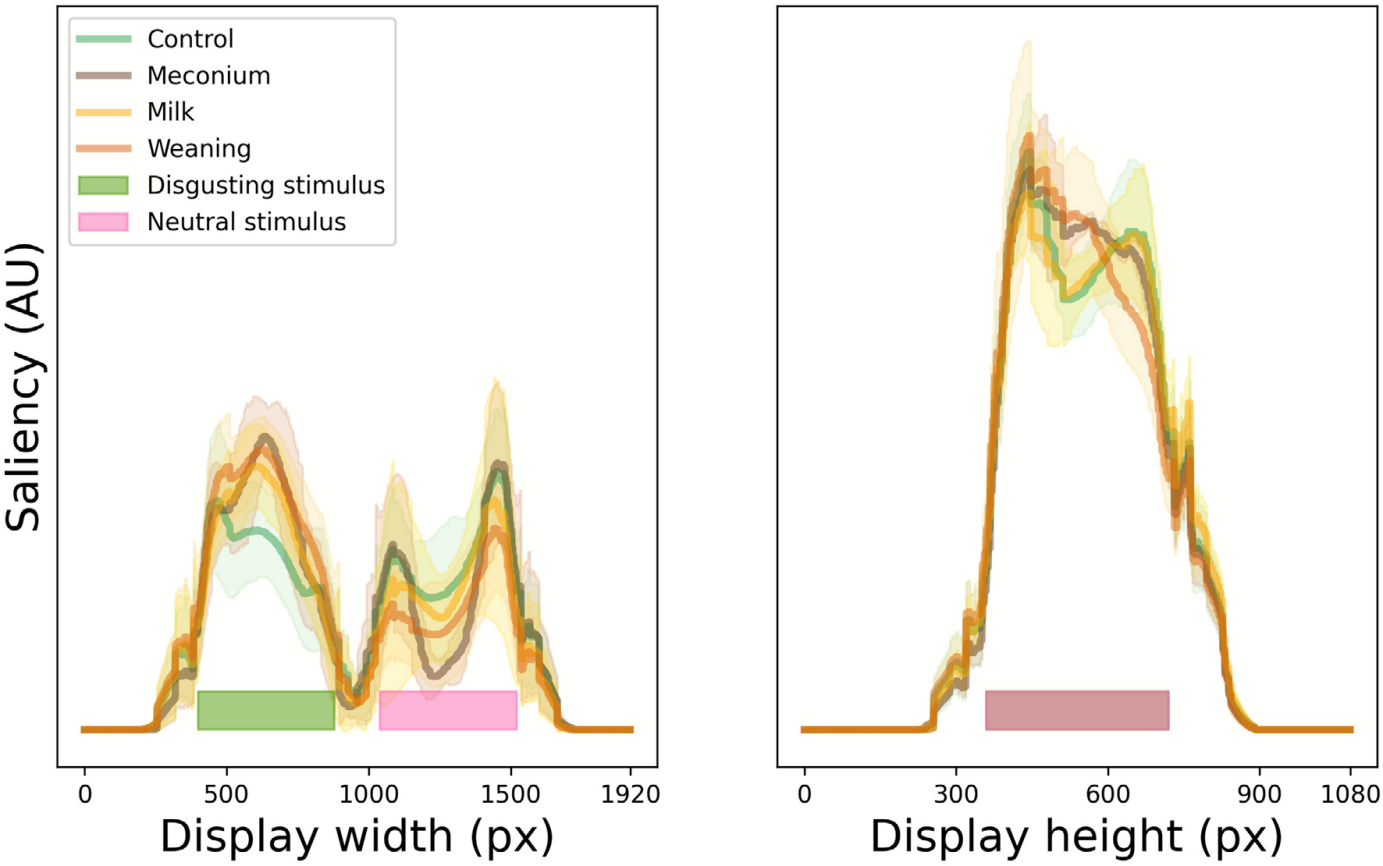
Visual saliency profiles of stimulus displays along the horizontal (left panel) and vertical axes (right panel). Averages across stimuli are shown as solid lines, with standard deviations indicated by shading. Four conditions are shown: General bodily effluvia unrelated to parenting (control, in green), meconium diaper stimuli (brown), diaper stimuli with feces from infants who were fed exclusively milk (yellow), and diaper stimuli with feces from children who had started or completed weaning (orange). For visualization purposes, disgusting stimuli are shown on the left, but this includes the mirrored results of displays where disgusting stimuli were shown on the right side of the display.

We computed low‐level visual saliency for each stimulus pair and setting (disgust left or disgust right) using color, intensity, and orientation channels from a popular model (Itti et al. 1998). Typically, this model uses many iterations of competition to inhibit all but the most salient areas, but we used only one such iteration to ensure better estimation of saliency across a display. Histograms of saliency profiles across the horizontal and vertical axes are shown in Figure 2. While variability was high, a bias in the direction of disgusting stimuli can be observed. This would predict an (early) attentional bias towards disgusting stimuli. A saliency‐weighted rendering of each individual stimulus display has been included in Appendix S1.

### 2.8 Data Reduction and Statistical Analysis

Self‐reported disgust sensitivity was computed as the averages (after reverse‐coding, where relevant) of the Disgust Scale—revised and the Parenting Domain Disgust Scale. Dwell time in each trial of the preferential looking task was computed as the proportion of time in a trial during which a participant hovered with their cursor over three areas of interest: the disgusting stimulus, the neutral stimulus, and the display outside of either.

Participants were divided into three main groups: control (non‐parents), parents to milk‐fed infants (those who reported their child exclusively consumed milk), and parents to weaning/weaned children (those who reported their children had started eating foods other than milk). The reasoning behind this grouping stems directly from our hypothesis about parental habituation to two qualitatively different types of infant feces. The change from mustard‐like to adult‐like feces occurs more or less immediately upon the introduction of solid foods, so parent participants were divided into those who exclusively milk‐fed their children and those who had started or completed the weaning process. For simplicity, we will refer to these groups as “pre‐weaning” and “post‐weaning”.

We used linear mixed‐effects models to analyze self‐report and behavioral data. We included a null model (i.e., only random effect and an intercept), simple models (e.g., only random effect and stimulus type as a fixed effect), and more complex models (e.g., using all factors enumerated in the next paragraph). The purpose of this was to compare the goodness of fit of each model, weighing both simplicity and accuracy in the goodness of fit.

Participant number was treated as a random effect. Fixed effects were group (control, pre‐weaning parent, post‐weaning parent), stimulus type (disgust or neutral), disgust type (general bodily effluvia, meconium diaper, milk‐poo diaper, weaned‐poo diaper), and stimulus repetition (first or second presentation). Within each model, we also included all possible interaction effects between included factors.

Linear mixed‐effects model fits were quantified by the Bayesian Information Criterion (BIC). The lowest BIC was defined as the best‐fitting model, and differences in fit were quantified as differences in BIC (ΔBIC) and as Bayes Factors (using Wagenmakers 2007). These were interpreted according to Raftery (1995) and an adjusted version of Jeffreys' guidelines (Andraszewicz et al. 2015; Jeffreys 1961), respectively. Concretely, this meant we considered ΔBIC values over 10 as very strong evidence; and Bayes Factors of 1–3 “anecdotal”, 3–10 as “moderate”, 10–30 as “strong”, 30–100 as “very strong”, and over 100 as “extreme” evidence for the alternative hypothesis (or its reciprocal as evidence for the null hypothesis).

Where relevant following linear mixed‐effects analyses, comparisons between subsets of the data were done using Welch's *t*‐test to account for any differences in size and variance between groups. Relationships between variables were assessed with bivariate correlations. Bayes Factors for t‐tests were computed using a Cauchy prior with scale factor 0.707 (Rouder et al. 2009), and using “Jeffreys exact” Bayes Factors (Ly et al. 2016) for correlations (using *κ* = 1).

The above is an established way of analyzing preferential looking data that has been applied to eye‐tracking (Dalmaijer et al. 2021; Nord et al. 2021) and MouseView.js data (Anwyl‐Irvine et al. 2022; Edgar et al. 2024). Analyses were conducted in Python 3.10.12, using NumPy 1.26.3 (Harris et al. 2020), SciPy 1.12.0 (Virtanen et al. 2020), statsmodels 0.14.1, and Pingouin 0.5.4 (Vallat 2018); and Matplotlib 3.8.2 (Hunter 2007) was used for data visualization.

## 3 Open Science

### 3.1 Open Materials

Stimuli produced for this study are available through Zenodo (doi.org/10.5281/zenodo.14515338). Anonymised data can also be found on Zenodo (https://doi.org/10.5281/zenodo.14450483), and the accompanying analysis scripts on GitHub (https://github.com/esdalmaijer/2024_parent_disgust). The experiment can be found on Gorilla (https://app.gorilla.sc/openmaterials/1113808).

### 3.2 Pre‐Registration

We did not formally pre‐register our study. We reported all variables that we recorded, which can be independently confirmed through the shared experiment. The hypotheses listed in the Introduction section were formulated a priori, as was the intention to divide the sample into a control, pre‐weaning, and post‐weaning group. Despite the lack of pre‐registration, some credibility for these statements can be derived from the fact that our predictions were not borne out by the data.

Exploratory analyses were also conducted. These are reported under the headers “Parents' age was unrelated to their disgust avoidance” and “Correlation between self‐reported disgust sensitivity and disgust avoidance”, and were the result of reviewer queries. The addition of these analyses can be independently verified through their respective appearances in versions 2 and 3 of the preprint of this manuscript (Huang et al. 2024).

## 4 Results

### 4.1 Manipulation Check: Sampled Parents' Diaper Changing Frequency

Table 1 shows diaper changing frequencies for parents across weaning stages. No parent reported never changing any diapers; almost all parents to diaper‐wearing children reported changing at least two diapers per day. Among parents to fully weaned children, 54% reported that none of their children wore diapers. There did not seem to be a gender difference in diaper changing frequency.

**TABLE 1:** Daily diaper changing frequency for parents. Data is split by parent gender and the weaning stage of their only or youngest child. Weaning stages are self‐reported and include children only drinking milk, children mostly on milk but started weaning, children drinking some milk but mostly weaned, or children being fully weaned. The N/A column reflects parents reporting that none of their children wear diapers. One non‐binary participant was omitted from the count to avoid deanonymisation.

	N/A	Never	< 1	1	2	3–5	6–10	11–15
Only milk	0	0	0	0	3	5	17	3
Men	0	0	0	0	2	2	3	0
Women	0	0	0	0	1	3	14	3
Mostly milk	1	0	0	0	1	3	10	1
Men	0	0	0	0	1	1	1	0
Women	1	0	0	0	0	1	9	1
Mostly weaned	0	0	0	1	2	7	1	0
Men	0	0	0	1	1	2	0	0
Women	0	0	0	0	1	5	1	1
Fully weaned	24	0	2	0	4	12	1	1
Men	4	0	1	0	3	4	0	0
Women	20	0	1	0	1	8	1	1

These figures illustrated that parents of diaper‐wearing children in our sample changed diapers with high frequency. This confirmed our assumption that parents of young children are frequently exposed to diapers, and thus validates testing disgust habituation in this sample.

### 4.2 Parents Self‐Reported Similar Disgust Sensitivity

There was no evidence for or against a difference in average scores on the revised Disgust Scale between controls [*M* = 2.12, SD = 0.53] and pre‐weaning parents [*M* = 2.44, SD = 0.61], *t*(48.85) = 1.03, *p* = 0.307, BF10 = 0.384; and moderate evidence against a difference between controls and post‐weaning parents [*M* = 2.08, SD = 0.57], *t*(110.68) = −0.89, *p* = 0.378, BF10 = 0.28. This suggests that post‐weaning parents were similar in self‐reported disgust sensitivity to controls and that no such determination could be made for pre‐weaning parents, but note that the weight of evidence is relatively low.

There was also no evidence for or against a difference in average scores on the parent‐specific extension to the Disgust Scale between controls [*M* = 2.11, SD = 0.81] and pre‐weaning parents [*M* = 1.81, SD = 0.67], *t*(64.37) = −1.84, *p* = 0.070, BF10 = 1.03; but there was strong evidence for lower average scores (compared to controls) in post‐weaning parents [*M* = 1.62, SD = 0.69], *t*(94.19) = −3.25, *p* = 0.002, *d* = 0.41, BF10 = 20.56. This suggests post‐weaning parents showed lower self‐reported disgust sensitivity specific to parenting situations compared to non‐parent controls, but that the weight of evidence is too low to make a similar determination for pre‐weaning parents.

**FIGURE 3:**
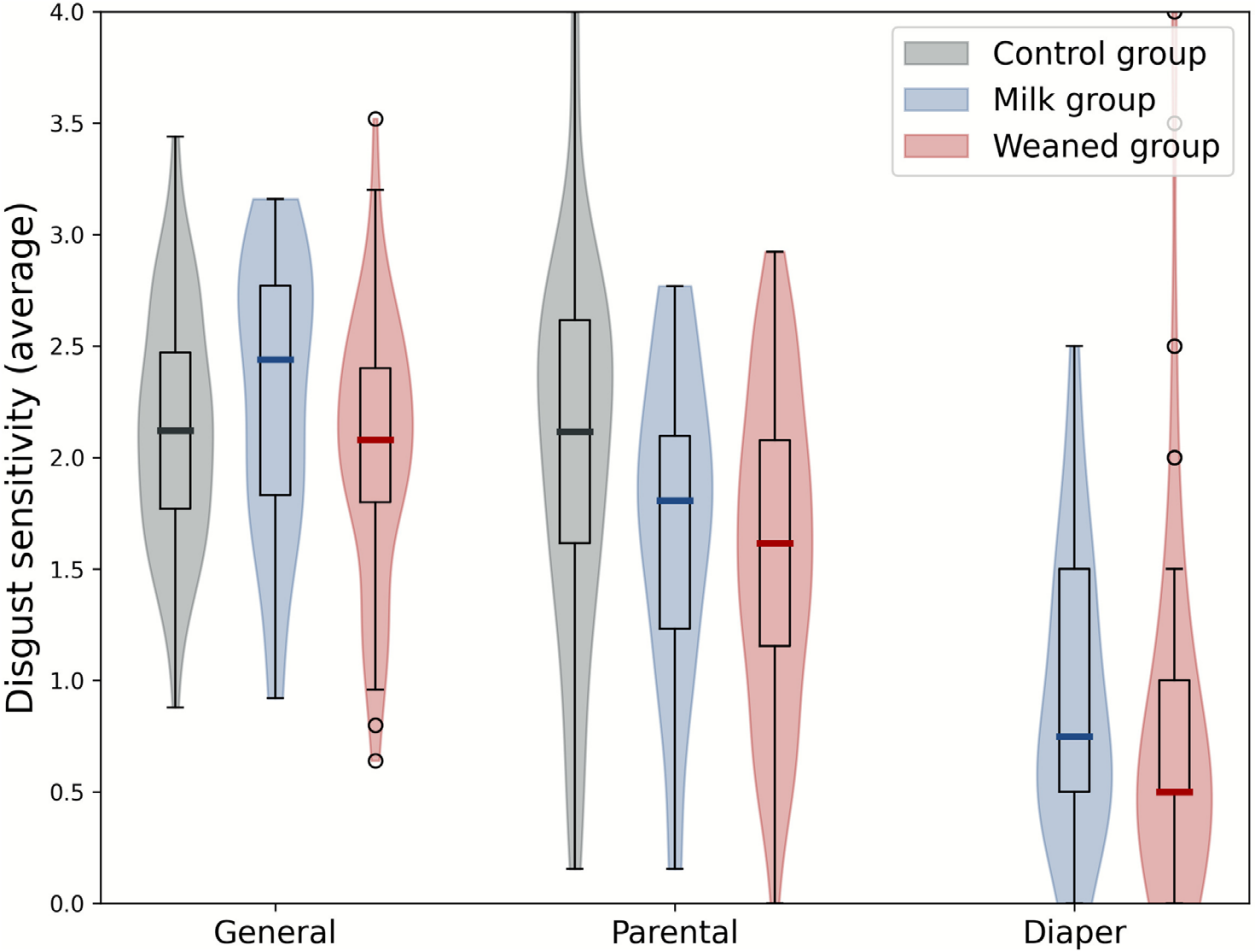
Average scores (*y*‐axis) on the Disgust Scale – revised (“General”), the extension to the Disgust Scale for parenting‐specific scenarios (“Parenteral”), and on self‐reported disgust to wet and/or soiled diapers (“Diaper”) from the parenting questionnaire. Violin and box plots reflect the distribution of scores of each group: Non‐parent controls (gray), parents whose (youngest) child is exclusively milk‐fed (blue), and parents to children who are weaning or have been fully weaned (red). The pre‐weaning group seems to show higher median disgust sensitivity, but such a difference was not apparent in statistical analysis (see main text).

Self‐reported disgust sensitivity scores are visualized in Figure 3. This figure also includes scores on the wet and soiled diaper disgust sensitivity question from the parent questionnaire, which is included for illustration and not analyzed (no control data were gathered for this question).

### 4.3 Parents Show Reduced Behavioral Disgust Avoidance

**FIGURE 4:**
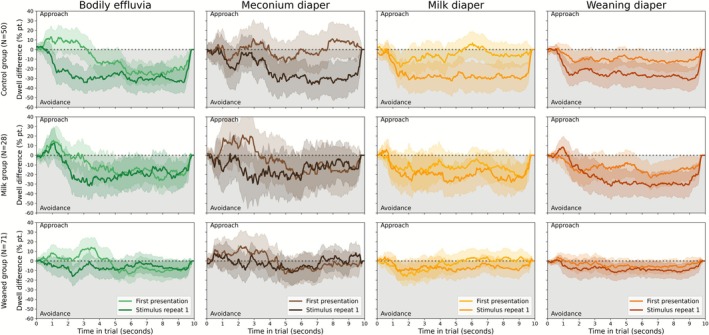
Average dwell time difference between disgust and neutral stimuli (*y*‐axis) to quantify participants' disgust approach (above dotted line) or avoidance (below dotted line) throughout a trial (*x*‐axis). The data are split between three groups: Non‐parent controls (top row), parents whose (youngest) children are exclusively milk‐fed (middle row), and parents to weaning or weaned children (bottom row). Each column reflects a specific disgust stimulus type: General bodily effluvia (green), diapers with meconium feces (brown), diapers with milk feces (yellow), and diapers with weaning/weaned feces (orange).

Disgust avoidance was quantified as the difference in dwell time between the neutral and the disgusting stimulus and is visualized in Figure 4. This figure shows a brief initial lack of disgust avoidance, followed by sustained avoidance throughout the trial across stimulus types. This was primarily true for the non‐parent control group and pre‐weaning parents and much less pronounced in post‐weaning parents. The statistics that accompany these observations are provided in the next paragraphs.

The outcome (dependent variable) for models described here was dwell time: the proportion of time in a trial during which a participant's cursor‐locked aperture was focused within an area of interest (i.e., over the neutral or disgusting stimulus). The best‐fitting linear mixed‐effects model [ΔBIC = 183.4, BF = 6.74e39, “extreme” evidence for this model over the next‐best] included group membership (control, pre‐weaning parents, or post‐weaning parents), stimulus type (disgust or neutral), and repetition (first or second presentation); as well as all possible interactions between these factors. This suggests that these three factors were most important to explain the data. The best‐fitting model most notably excludes the factor of disgust type, suggesting that general bodily effluvia and diapers with different contents (meconium, milk‐only feces, or weaning feces) were avoided to similar extents.

This model showed no effect of being in the pre‐weaning parent group [*β* = −0.099, 95% CI (−0.290, 0.093), *t* = −1.01, *p* = 0.314] or the post‐weaning parent group [*β* = 0.021, 95% CI (−0.129, 0.171), *t* = 0.28, *p* = 0.782] compared to control; and a main effect of stimulus [*β* = 0.319, 95% CI (0.249, 0.388), *t* = 9.00, *p* = 8.88e‐16]. This suggests overall dwell time was equivalent between groups, but that dwell time was higher for neutral than for disgusting stimuli. This is as expected, and confirms that participants (on average) showed behavioral disgust avoidance.

There was also a main effect of repetition [*β* = −0.456, 95% CI (−0.526, −0.387), *t* = −12.89, *p* < 1e‐16], no interaction effect between repetition and pre‐weaning group membership [*β* = 0.049, 95% CI (−0.067, 0.165), *t* = 0.83, *p* = 0.407], and an interaction effect between repetition and post‐weaning group membership [*β* = 0.161, 95% CI (0.070, 0.251), *t* = 3.48, *p* = 6.51e‐4]. There were also interaction effects between pre‐weaning group membership, stimulus type, and stimulus repetition [*β* = −0.329, 95% CI (−0.493, −0.166), *t* = −3.94, *p* = 1.24e‐4], and between post‐weaning group membership, stimulus type, and stimulus repetition [*β* = −0.496, 95% CI (−0.624, −0.368), *t* = −7.59, *p* = 3.33e‐12]. This suggests dwell times for both stimuli were lower in the second compared to the first presentation (i.e., participants spent more time hovering their cursor over neither stimulus), and that this tendency was the same between the control group and pre‐weaning parents. However, post‐weaning parents showed a lower reduction of stimulus dwell time on the second presentation of each stimulus compared to the control group. In addition, pre‐weaning and post‐weaning parents showed smaller differences in disgust avoidance (dwell time difference between neutral and disgusting stimuli) over the stimulus repetitions compared to controls.

Crucially, there were interaction effects between pre‐weaning parent group membership (compared to controls) and stimulus [*β* = 0.142, 95% CI (0.026, 0.257), *t* = 2.40, *p* = 1.77e‐2], and between post‐weaning parent group membership (compared to controls) and stimulus [*β* = −0.196, 95% CI (−0.286, −0.105), *t* = −4.24, *p* = 3.97e‐5]. This suggests that, compared to controls, pre‐weaning parents had a stronger bias towards the neutral stimulus (i.e., stronger disgust avoidance). On the other hand, post‐weaning parents differed from controls in the opposite way: they had a stronger bias towards the disgusting stimulus (i.e., reduced disgust avoidance).

### 4.4 Parents' Age Was Unrelated to Their Disgust Avoidance

The following analyses relating to parental age and temporal distance to active diaper changing were exploratory: they were conducted not to address an a priori hypothesis, but rather in response to expert feedback on a prior version of this article.

Some of the parents included in the previous analysis no longer actively changed diapers, because their children had grown out of them (Table 1). These “distal” parents were no different in their behavioral avoidance (dwell‐time difference between neutral and disgusting stimuli) of bodily effluvia than the “proximal” parents who still regularly changed diapers [*t*(40.85) = −0.47, *p* = 0.643, BF10 = 0.26, “moderate” evidence against a difference]. The same was true for diaper‐specific stimuli [*t*(42.57) = −0.00, *p* = 0.999, BF10 = 0.24, “moderate” evidence against a difference].

There was also moderate evidence against a correlation between age and disgust avoidance for bodily effluvia for proximal [*R*(73) = −0.06, *p* = 0.583, BF10 = 0.17] and distal parents [*R*(24) = −0.24, *p* = 0.243, BF10 = 0.47, “anecdotal” evidence], nor for diaper stimuli for proximal [*R*(73) = −0.07, *p* = 0.535, BF10 = 0.17] or distal parents [*R*(24) = −0.10, *p* = 0.639, BF10 = 0.28].

There was moderate evidence for a correlation between age and disgust avoidance for the non‐parent (control) group in the bodily effluvia condition [*R*(49) = −0.36, *p* = 0.010, BF10 = 4.49, “moderate” evidence], but no evidence for or against this for diaper stimuli [*R*(49) = −0.25, *p* = 0.081, BF10 = 0.77]. This suggests that disgust avoidance could be lower in older age in non‐parents, at least for some stimuli.

For linear mixed‐effects models of dwell time, including age improved [ΔBIC = 20.7, BF = 3.05e4, “extreme” evidence for this model over the next‐best] the best‐fitting model and resulted in a model with fixed effects age (standardized age in years), group (control, pre‐weaning parent, or post‐weaning parent), stimulus (disgust or neutral), and repetition (first or second presentation) and all possible interactions between them. This generally replicated the findings from the model without age, with the exception that the interaction between pre‐weaning group membership and stimulus was not statistically significant [*β* = 0.115, 95% CI (−0.016, 0.247), *t* = 1.72, *p* = 0.088], suggesting that the previously identified tendency for pre‐weaning parents to avoid disgusting stimuli more than non‐parents could have been partially driven by age. There was also an effect of age [*β* = 0.106, 95% CI (0.007, 0.205), *t* = 2.10, *p* = 0.037], an interaction between age and repetition [*β* = 0.103, 95% CI (0.043, 0.162), *t* = 3.39, *p* = 9.01e‐4], suggesting that older individuals had more dwell time over either stimulus (i.e., less dwell outside areas of interest) and that this increased with stimulus repetition.

There was an interaction between age and stimulus type [*β* = −0.140, 95% CI (−0.200, −0.081), *t* = −4.64, *p* = 7.73e‐6], and an interaction between age, stimulus type, and repetition [*β* = −0.229, 95% CI (−0.313, −0.145), *t* = −5.34, *p* = 3.36e‐7]; suggesting that older participants showed lower avoidance of disgusting stimuli, especially when stimulus pairs were repeated. There were further interactions between age, stimulus type, and membership of the post‐weaning parent group [*β* = −0.101, 95% CI (0.016, 0.187), *t* = 2.33, *p* = 0.021]; between age, repetition, and membership of the post‐weaning parent group [*β* = −0.172, 95% CI (−0.258, −0.087), *t* = −3.96, *p* = 1.17e‐4]; and between age, stimulus type, stimulus repetition, and membership of the post‐weaning parent group [*β* = 0.328, 95% CI (0.207, 0.448), *t* = 5.32, *p* = 3.77e‐7]. These suggested that, compared to post‐weaning parents, participants in the non‐parent control group were less avoidant of disgusting stimuli as a function of age, and that this increased with stimulus repetition.

In sum, disgust avoidance decreased with age for non‐parents. However, this effect did not extend to parents, even those who no longer changed diapers.

### 4.5 Gender Effect on Disgust Avoidance?

Inclusion of gender in the best‐fitting model of dwell time resulted in a substantially worse fit [ΔBIC = 86.5, BF = 6.11e18, “extreme” evidence for the model without gender]. This model offers no statistically significant main or interaction effects that included gender, with the sole exception of an interaction effect of stimulus, gender (not being a man), and being in the pre‐weaning group [*β* = −0.436, 95% CI (−0.701, −0.170), *t* = −3.21, *p* = 1.60e‐3]. Without further context, this would suggest that the increase in disgust avoidance in pre‐weaning parents (compared to non‐parent controls) was different for fathers. However, the model that did not include gender fit convincingly better, which could be taken as evidence against a gender effect.

This finding replicates earlier robust null effects of sex or gender on behavioral disgust avoidance in adults (Dalmaijer et al. 2021) and children (Alladin et al. 2024).

### 4.6 Correlation Between Self‐Reported Disgust Sensitivity and Disgust Avoidance

We computed behavioral disgust avoidance as the difference in dwell time for the neutral and disgusting stimuli, with negative values indicating disgust avoidance (same direction as Figure 4). Avoidance of general bodily effluvia correlated with self‐reported disgust sensitivity (average score on the revised Disgust Scale) for parents [*R* = −0.28, *p* = 0.004, BF10 = 6.80] and non‐parent controls [*R* = −0.37, *p* = 0.008, BF10 = 5.65] (both “moderate” evidence for an association).

There was also evidence for associations between self‐reported disgust sensitivity and avoidance of diapers with meconium [*R* = −0.44, *p* = 0.001, BF10 = 26.34, “strong” evidence], milk feces [*R* = −0.37, *p* = 0.008, BF10 = 5.11, “moderate” evidence], and weaning feces [*R* = −0.41, *p* = 0.003, BF10 = 13.34, “strong” evidence]. Parents showed lower associations for diapers with meconium [*R* = −0.16, *p* = 0.107, BF10 = 0.45], milk feces [*R* = −0.19, *p* = 0.064, BF10 = 0.68], and weaning feces [*R* = −0.20, *p* = 0.047, BF10 = 0.87]; all with Bayes Factors considered “anecdotal” evidence (i.e., not in favor of the null hypothesis).

These findings suggest that higher disgust sensitivity is associated with higher behavioral disgust avoidance for general bodily effluvia. For parenting‐specific stimuli, we found similar associations for non‐parents. However, parents showed lower associations, to the point that evidence for their existence was inconclusive.

## 5 Discussion

Our main finding is that parents showed substantially lower behavioral disgust avoidance than non‐parents. We think this is a consequence of repeated exposure to bodily effluvia, which parents in our sample received through frequent diaper changing. Unexpectedly, we found that pre‐weaning parents' children showed no such reduction in disgust avoidance. Further, our findings indicated minor evidence against a difference in general self‐reported disgust sensitivity between parents and non‐parents, but a lower self‐reported disgust sensitivity for parenting‐specific scenarios in post‐weaning parents.

Previous work found only small effects of parenthood status on self‐reported disgust sensitivity (Prokop and Fančovičová 2016; Stefanczyk et al. 2024), which is mirrored in our limited evidence for or against such an effect. We did find a moderate effect of parent status on self‐reported disgust sensitivity for parent‐specific situations, but only those with weaning or weaned children. This suggests that disgust habituation does occur in parents, but that it is of limited impact on self‐reported general disgust sensitivity.

On the other hand, post‐weaning parents showed much lower avoidance of parent‐specific stimuli (diapers with feces) and general bodily effluvia (not specifically associated with parenting). This confirmed our hypothesis that long‐term disgust habituation occurs in parents, presumably due to their frequent exposure to disgusting stimuli. It also confirmed that disgust habituation generalizes from parent‐specific stimuli to other bodily effluvia.

We were surprised to find no such effects among pre‐weaning parents. While we anticipated a specific habituation to diapers with milk feces and not to those with weaned feces, we instead found that pre‐weaning parents showed pronounced avoidance for all types of disgust stimuli, with a bias that was *stronger* than in non‐parent controls. This pattern of findings aligns with compensatory prophylaxis, which is discussed below. We hasten to point out the small size of this subset of our parent sample, which should limit confidence in this specific finding.

### 5.1 Evidence for Disgust Habituation With Limited Generalization

Our results align with reports of reduced behavioral disgust avoidance in care home workers (Edgar et al. 2024), and with self‐reported disgust as a function of exposure in medical (Reynolds et al. 2019; Rozin 2008; Schnapp et al. 2019) and non‐medical environments (Piazza et al. 2021; Prokop and Fančovičová 2016; Wabnegger and Schienle 2025). They also corroborate the previous study of time‐in‐job and reduction in self‐reported disgust for meat products in butchers and deli workers (Piazza et al. 2021).

Most previous research could have been subject to potential confounds through selection and survivorship bias, as it specifically focused on adaptations to professional exposure to disgust. The notion that individuals who are less disgust sensitive might be more likely to choose employment that will subject them to more disgust elicitors is borne out in medicine, where they are more likely to end up in for example, nursing or surgery (Consedine, Yu, Hill, and Windsor 2013; Consedine, Yu, and Windsor 2013; Consedine and Windsor 2014). A similar process could (partially) explain the correlation with time‐in‐job and self‐reported disgust sensitivity among butchers and deli workers (Piazza et al. 2021) or care home workers (Edgar et al. 2024): more disgust‐sensitive individuals might quit at a higher rate, which could appear as disgust habituation in cross‐sectional data.

Our results bypass this limitation by focusing on parents: the decision to attempt to conceive or to adopt is highly complex, and it is unlikely to be significantly impacted by disgust sensitivity. In addition, it is less feasible to quit being a parent due to repeated exposure to diapers than it would be to leave a job. We thus think that our findings offer additional and stronger support for the idea that long‐term disgust habituation does indeed occur.

In addition to the existence of long‐term disgust habituation, our results support the idea that it generalizes to elicitors beyond the narrow set of stimuli one habituated to. Past research has suggested this does not occur, for exmaple, in medical students whose specific habituation to cold dead bodies during anatomy classes did not extend to warm bodies (Rozin 2008). A conflicting finding suggested that care home workers did show generalization to core (bodily effluvia) and gore (body envelope violations and the results thereof) disgust elicitors only found outside their place of work (Edgar et al. 2024). We found that parents showed reduced avoidance of not only diapers but also general bodily effluvia, and thus our findings align with generalization.

There is a potential affective‐motivational reason for reduced disgust avoidance in parents (and other caregivers), as their frequent disgust elicitors are associated with children (or patients) in their care. Indeed, mothers are less disgusted by feces from their own infants (Case et al. 2006). If this were the only reason for reduced disgust avoidance, one would not expect it to generalize to diapers from other children (as used in this study) nor to other elicitors (as used in the control condition in this study). Hence, while motivated attention might play a role in reducing avoidance to disgust elicitors produced by the objects of our compassion, the longer‐term and generalized reduction in avoidance shown here and in past work (Edgar et al. 2024) is likely a consequence of habituation during continued exposure.

### 5.2 Evidence for Compensatory Prophylaxis?

An unexpected finding in our study was that pre‐weaning parents were more disgust‐avoidant than controls. This group even included milk‐feeding parents with previous children, so they could already be habituated due to prior experience of weaning diapers.

The fact that pre‐weaning parents are more disgust‐averse seems to align with the compensatory prophylaxis hypothesis. This theory suggests that individuals who are more sensitive to infection, or who would face a larger cost when infected, are more sensitive to disgust. One example of this is pregnancy, during which individuals indeed show higher disgust sensitivity (Fessler et al. 2005) that seems to correlate with blood markers of immunocompetence (Kaňková et al. 2022). There might even be a small increase in disgust sensitivity 6–14 weeks after giving birth (Dlouhá et al. 2023). Important context to this is that large longitudinal studies have found no changes in disgust sensitivity across women's menstrual cycle (Jones et al. 2018; Stern and Shiramizu 2022), so the compensatory prophylaxis hypothesis is not uniformly supported.

Increased disgust avoidance in parents during the first 6 months after birth (until weaning starts) has face validity. However, their children might actually be at lower immunocompetence between ages 1 to 5, when maternal antibodies (in utero and from breast milk) are no longer available and their own immune system is still immature (Simon et al. 2015). In fact, one could interpret our results as support for an opposite hypothesis: deliberate immune activation. Children lack a self‐reported sensitivity to disgust until age 5–7 years (Stevenson et al. 2010), although they do already show adult‐like behavioral avoidance at age 5 and over (Alladin et al. 2024). It could be argued that their parents' reduction in disgust avoidance serves the purpose of allowing children to come into contact with disease vectors to calibrate their immune system. However, it is rather unlikely that this would be a long‐standing evolutionary adaptation given the high levels of child mortality even in modern hunter‐gatherer societies (Gurven and Kaplan 2007). Computational work suggests that disgust‐approach is only selected for in environments where food is scarce, contamination levels are high, and contamination risks are low (Dalmaijer and Armstrong 2020).

Crucially, the sub‐sample of pre‐weaning parents was relatively small. Hence, our accidental finding that they show no reduction in disgust avoidance does not carry substantial evidential weight. Future research could remedy this.

## 6 Conclusion

Parents are faced with a large number of disgusting stimuli, including used diapers. Presumably as a consequence, they show markedly reduced behavioral avoidance of diapers with feces, but only after a period of increased disgust avoidance while their infant is exclusively fed milk. This lack of avoidance generalizes to bodily effluvia that are not associated with children. This suggests that while disgust is cognitively impenetrable and shows no short‐term habituation, individuals do habituate over longer periods of continued exposure.

## Author Contributions

Conceptualisation: Y.H., T.A., E.S.D. Data curation: E.S.D. Formal analysis: E.S.D. Investigation: Y.H., E.S.D. Methodology: Y.H., T.A., E.S.D. Software: E.S.D. Resources: I.E.D.‐D., J.A.D.‐D. Visualization: E.S.D. Writing – original draft: Y.H., E.S.D. Writing – review and editing: Y.H., I.E.D.‐D., J.A.D.‐D., T.A., E.S.D.

## Funding

The authors have nothing to report.

## Conflicts of Interest

The authors declare no conflicts of interest.

## Supporting information


**Appendix S1:** sjop70069‐sup‐0001‐AppendixS1.pdf.

## Data Availability

The data that support the findings of this study are openly available in Zenodo at https://doi.org/10.5281/zenodo.14450483, reference number https://doi.org/10.5281/zenodo.14450483.
