# Supplementary material for: Parents Develop Long‐Term Disgust Habituation, but Only After Beginning to Wean Their Children
Source: Scand J Psychol. 2026 Jan 6;67(3):787–800. doi: 10.1111/sjop.70069 (PMC13159516; doi:10.1111/sjop.70069)

Supplementary Information

Parents develop long-term disgust habituation, but only after weaning their children

Yifan Huang [1], Ivo E. Dalmaijer-Denning [2], Joris A. Dalmaijer-Denning [2], Thomas Armstrong [3], & Edwin S. Dalmaijer [1,2]

Affiliations

1. School of Psychological Science, University of Bristol, United Kingdom
2. University Day Nursery, University of Bristol, United Kingdom
3. Department of Psychology, Whitman College, Walla Walla, United States

Contact Details

Dr Edwin S. Dalmaijer, School of Psychological Science, University of Bristol, 12A Priory Road, Bristol, BS8 1TU, United Kingdom. edwin.dalmaijer@bristol.ac.uk

Saliency maps

The procedure behind how saliency was derived can be found in the main manuscript. Included here are examples of stimulus displays weighted by the low-level visual saliency computed for each. For consistency, examples show a stimulus pair with its disgusting stimulus on the left and its neutral on the right. The examples are blurred as they would be through the MouseView.js interface.

The original stimulus background was white, with images presented on the left and the right of the centre. (See main manuscript for details on size and placement.) In the following figures, areas of low visual saliency are depicted as dark, and areas of high visual saliency show the original stimulus.

Control stimuli

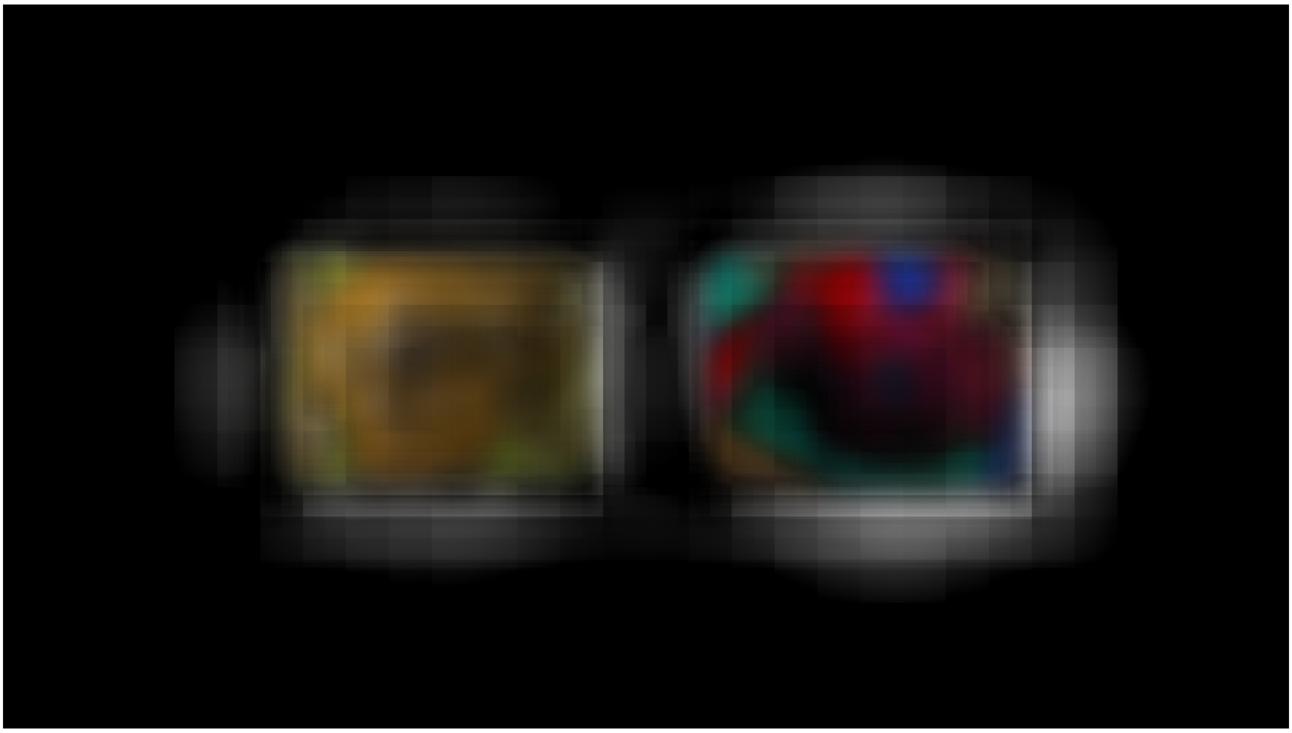

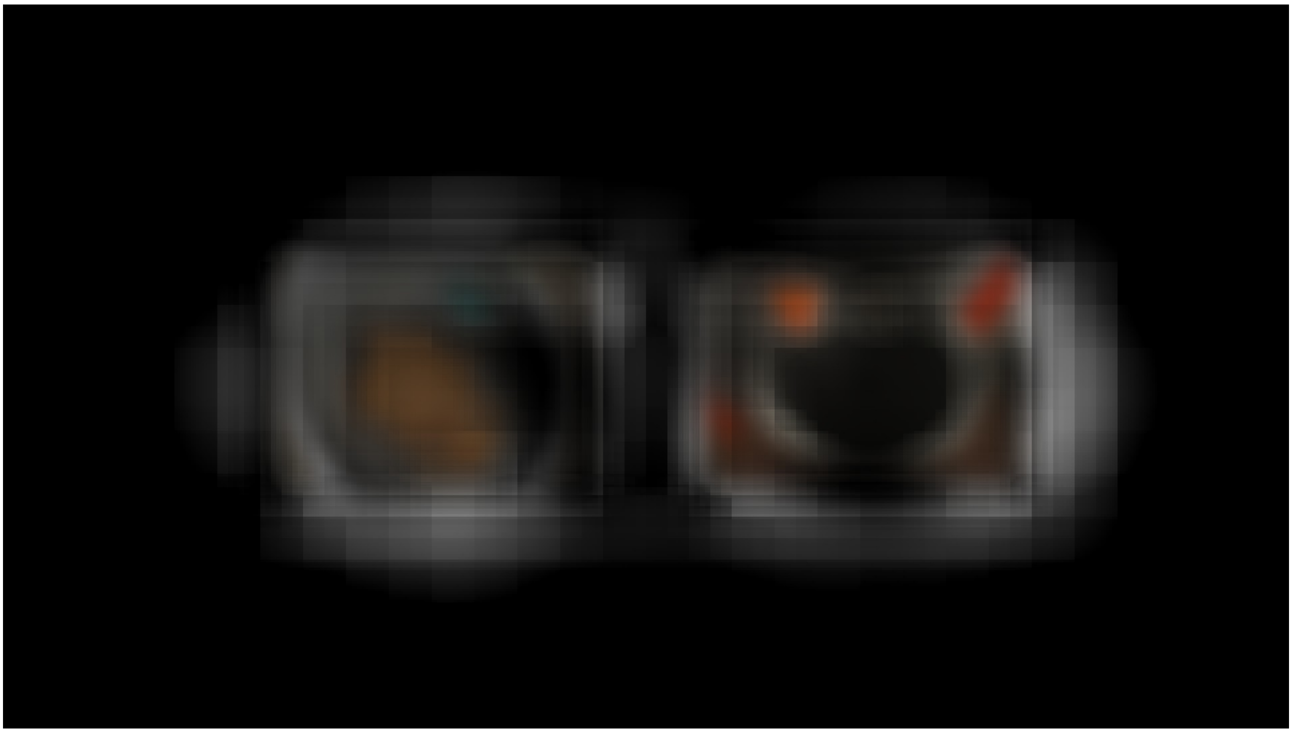

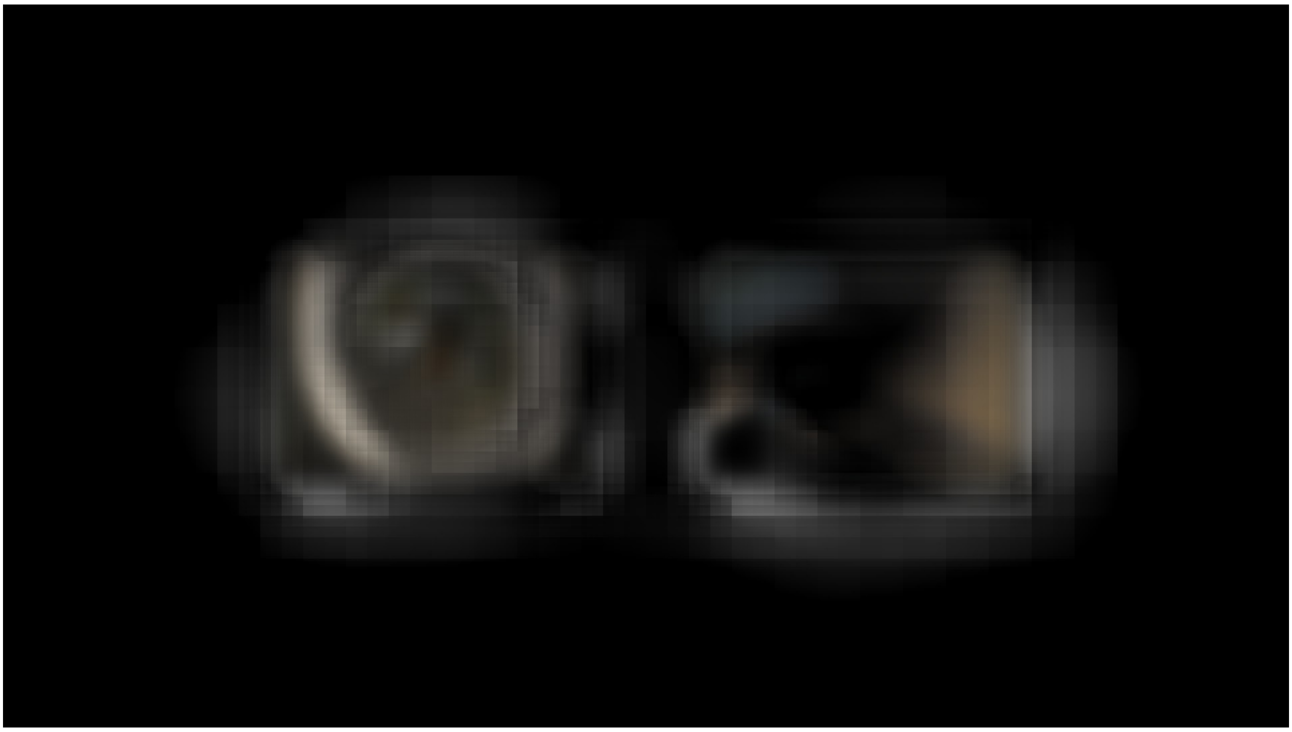

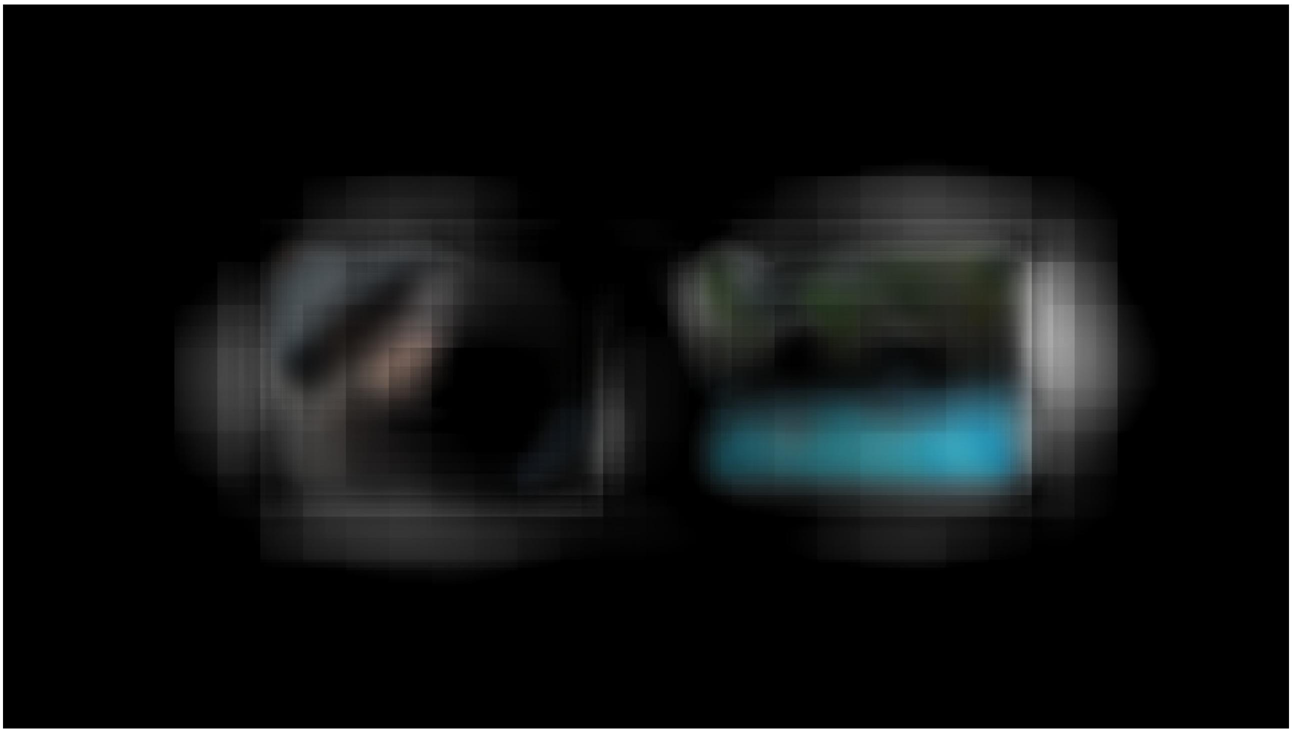

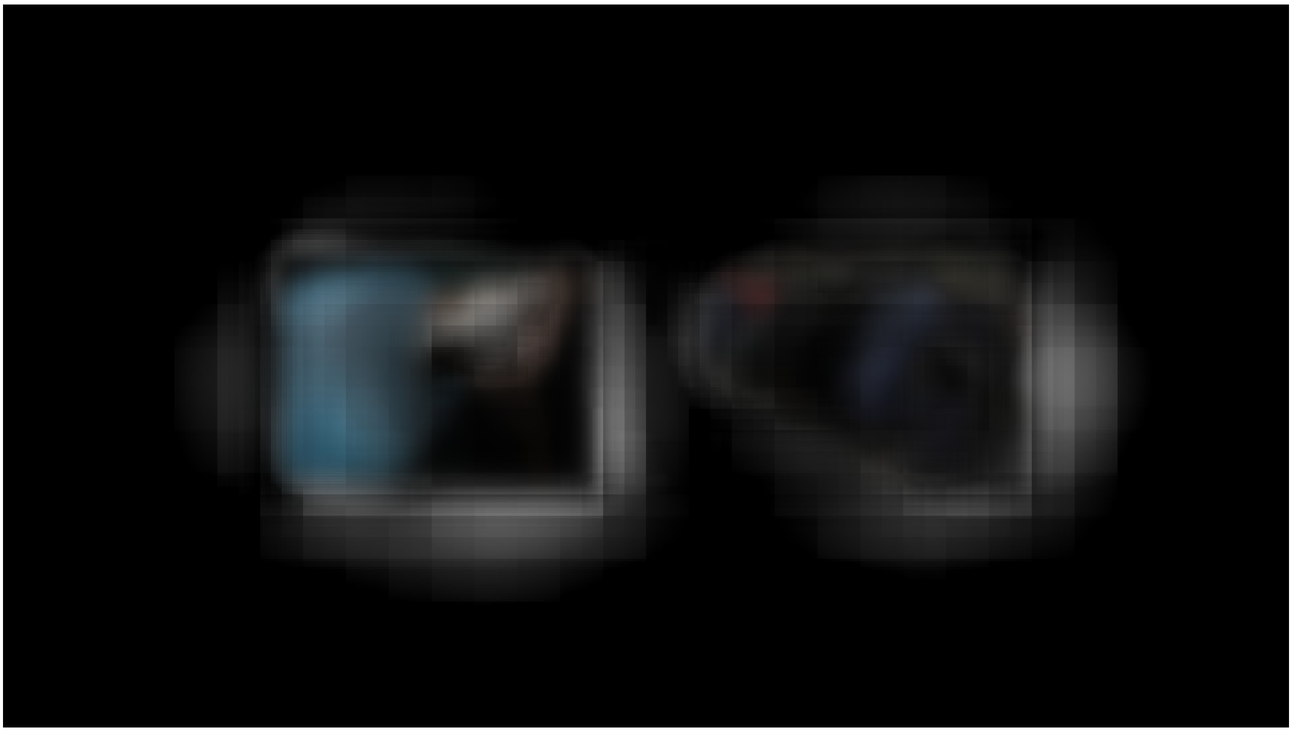

Meconium stimuli

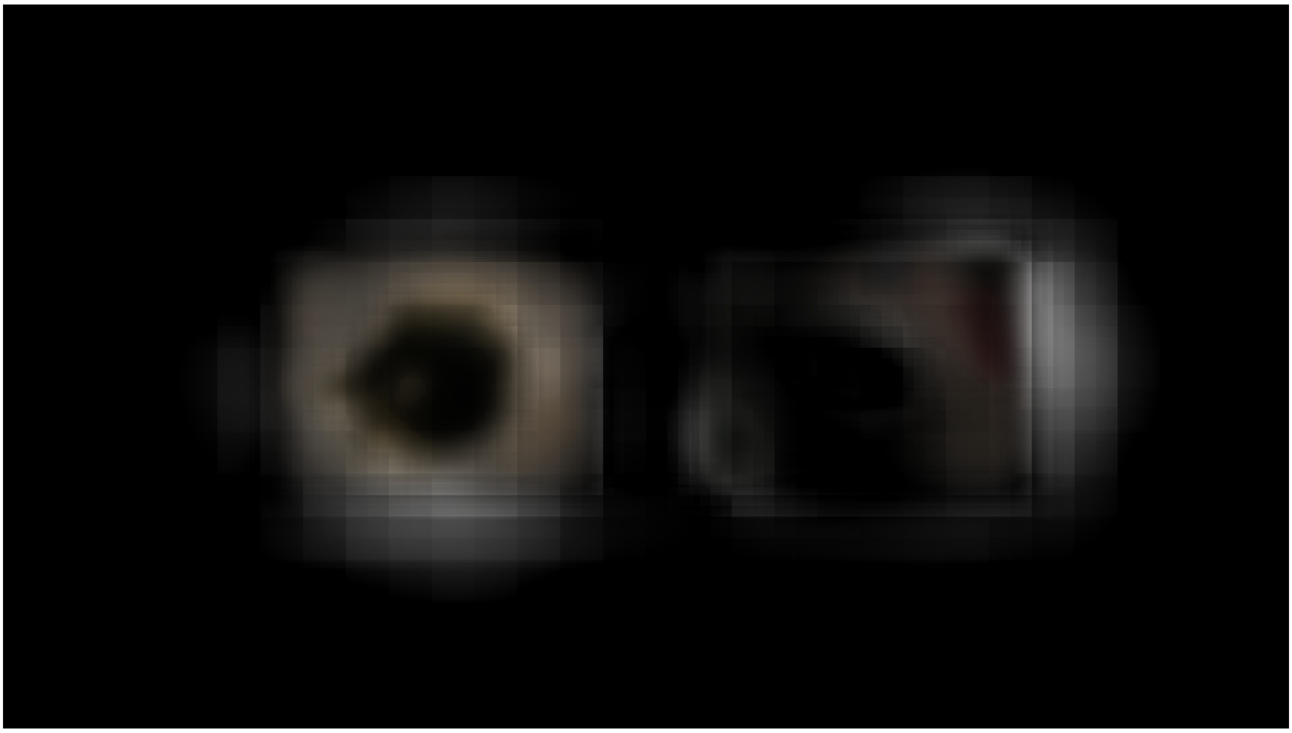

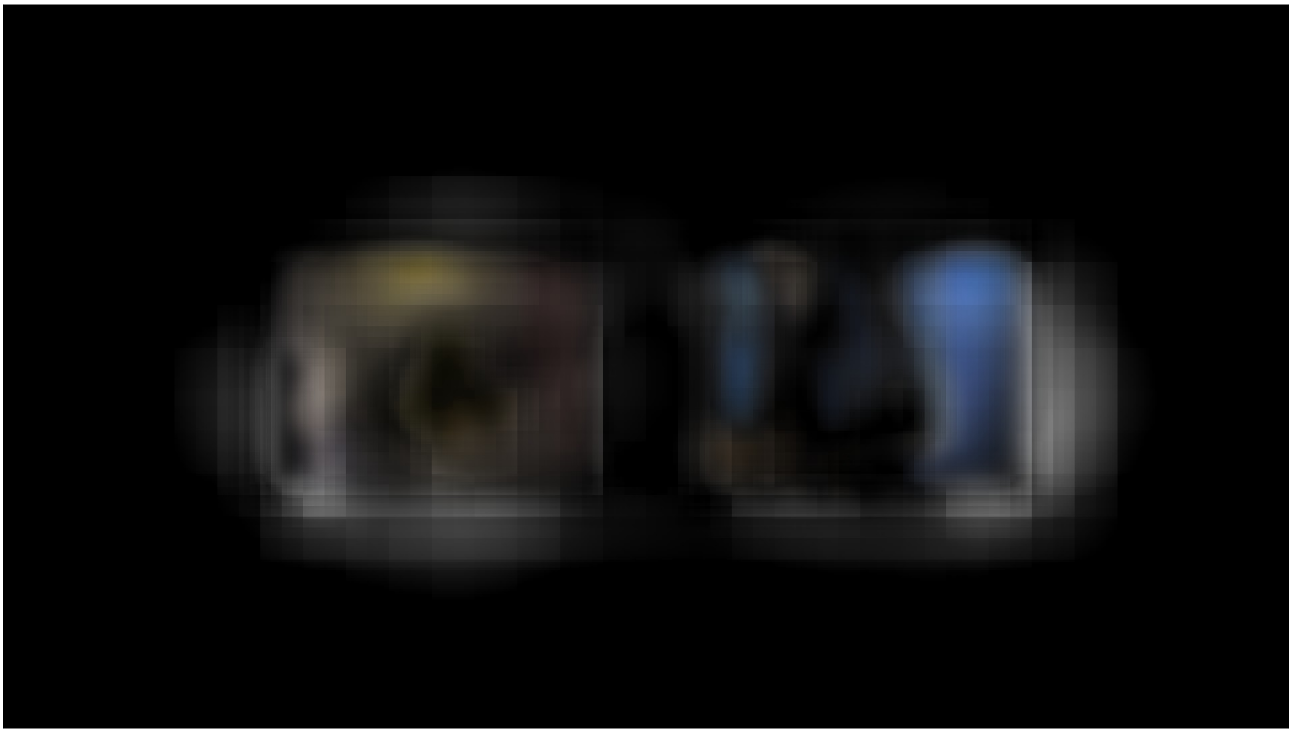

Milk faeces stimuli

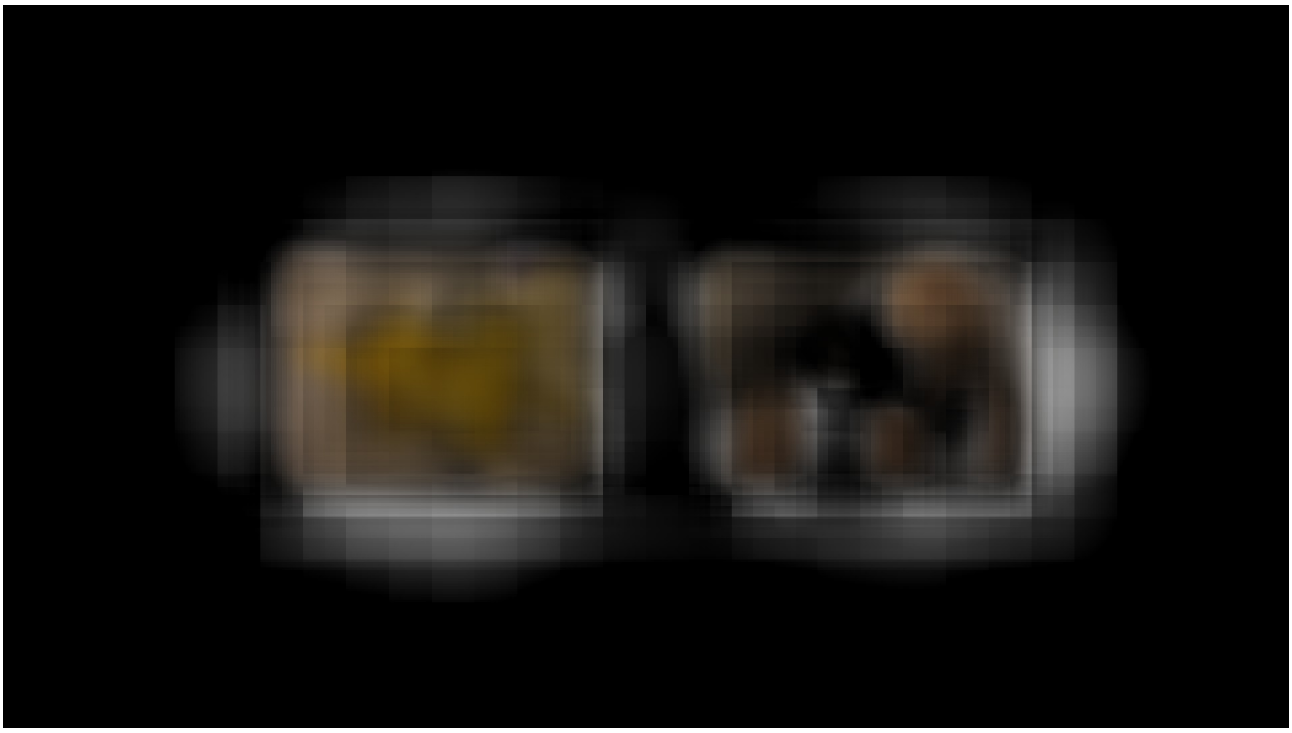

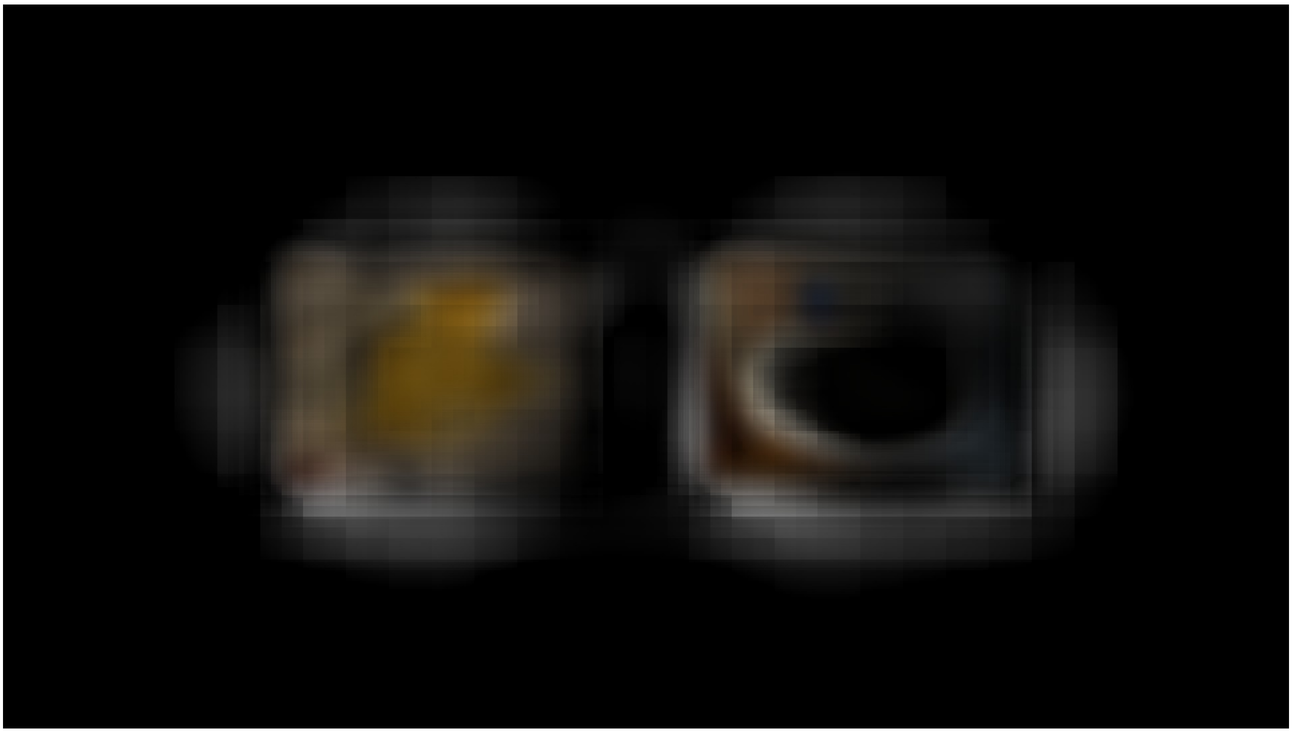

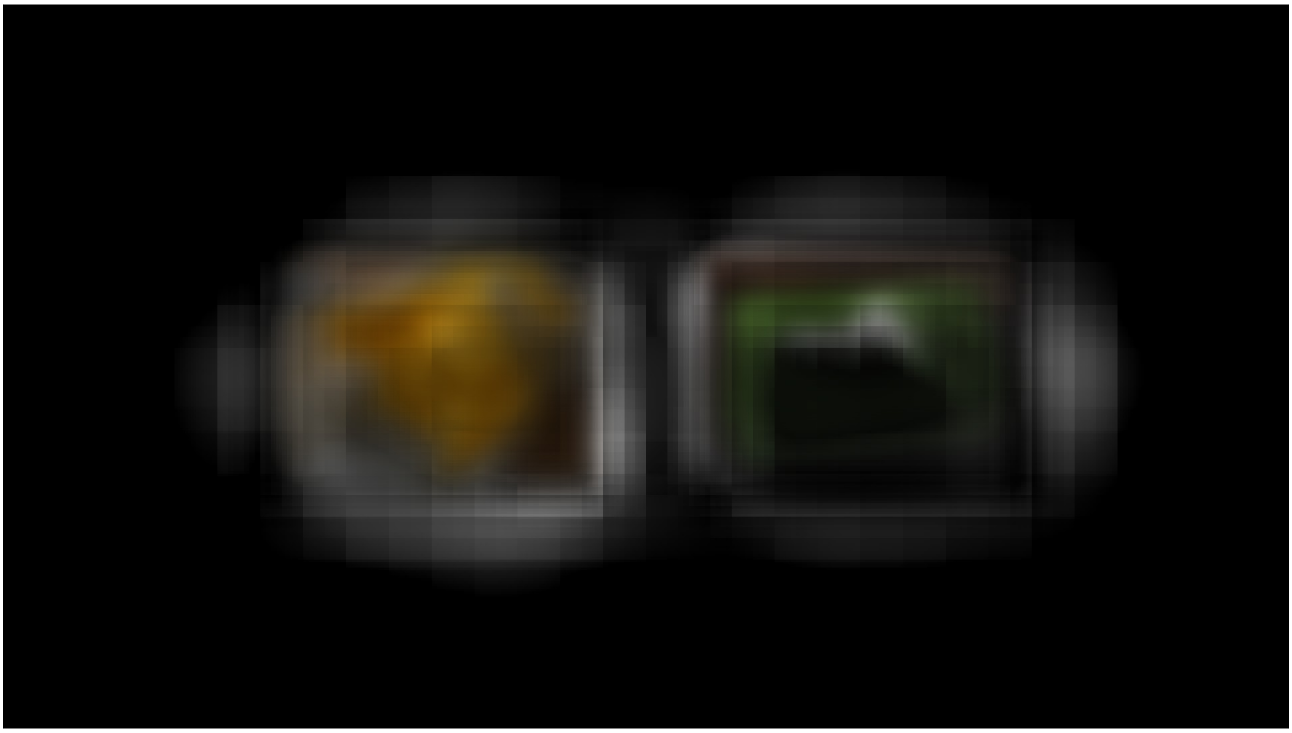

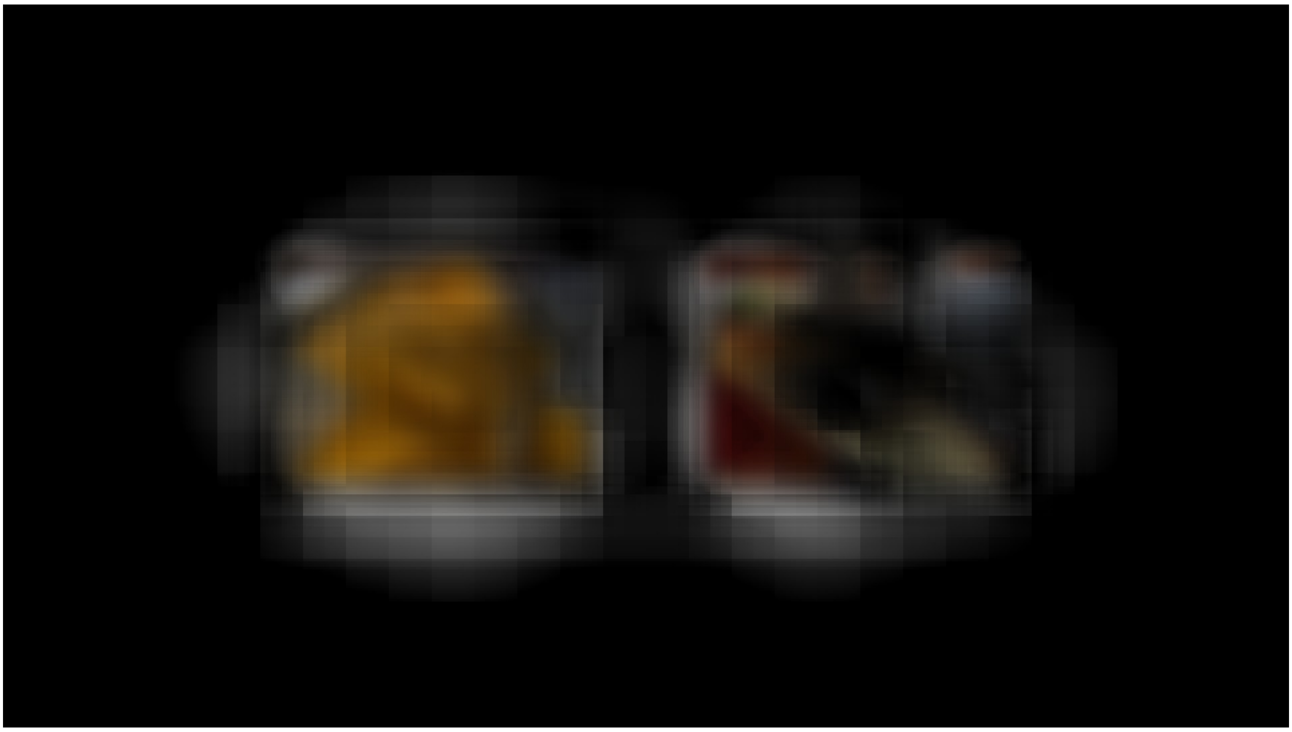

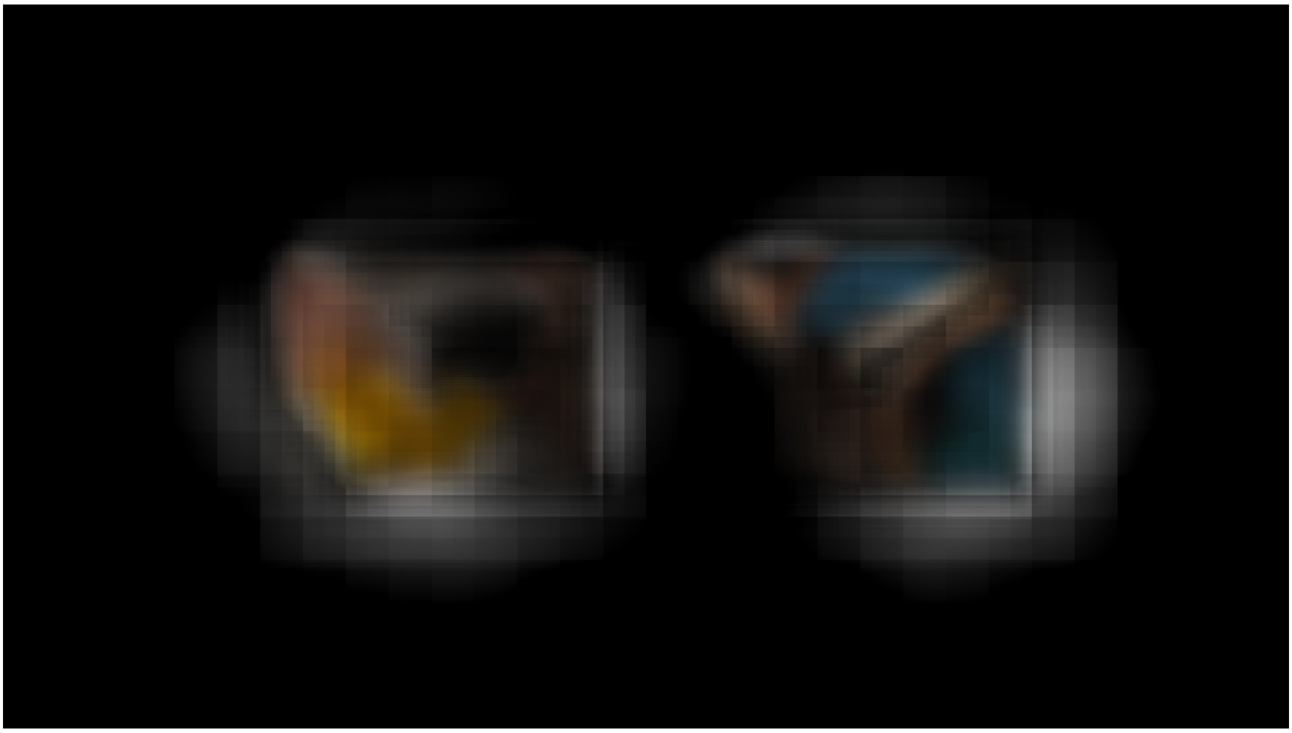

Weaning/weaned faeces stimuli

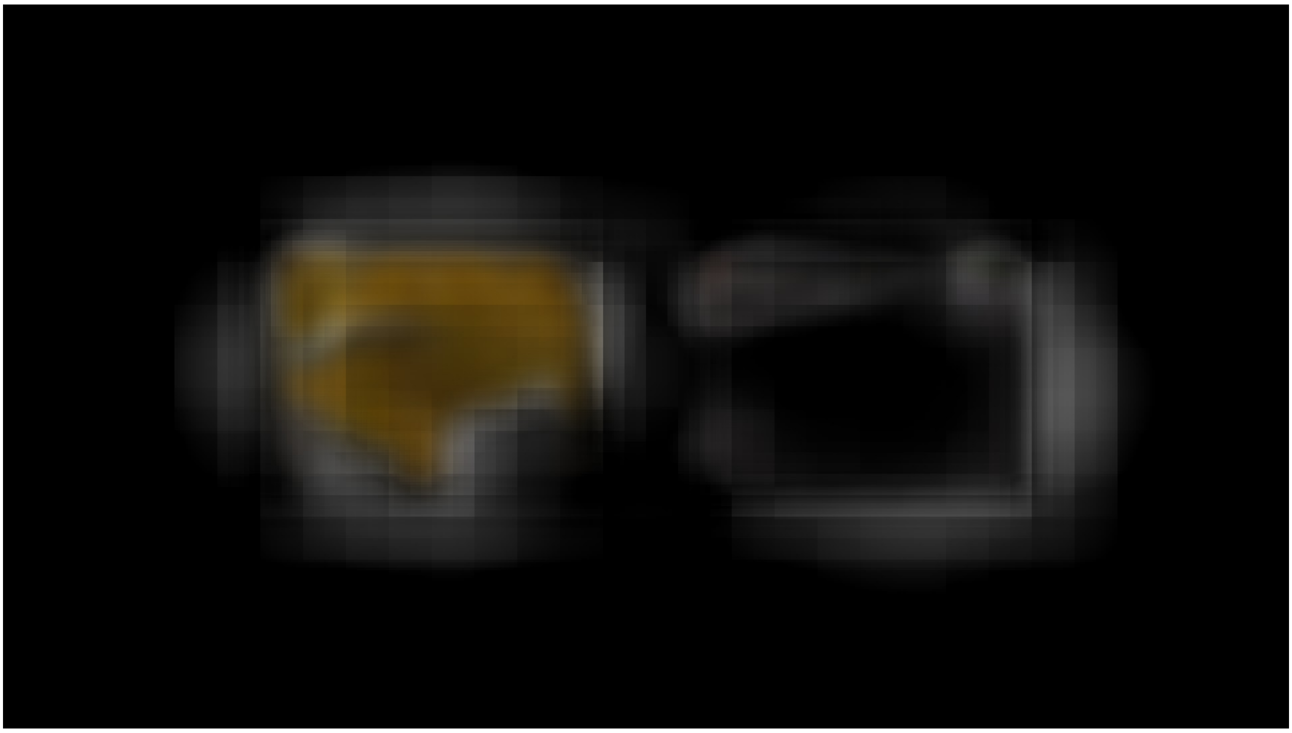

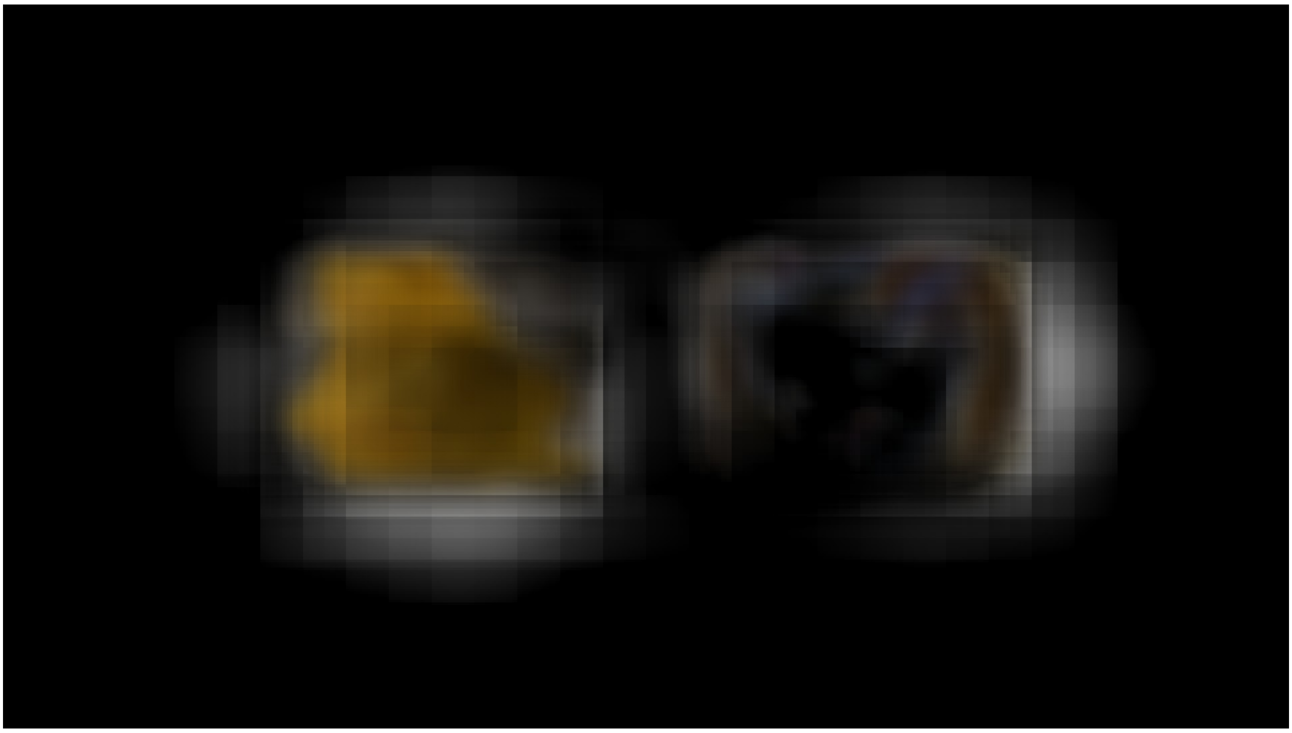

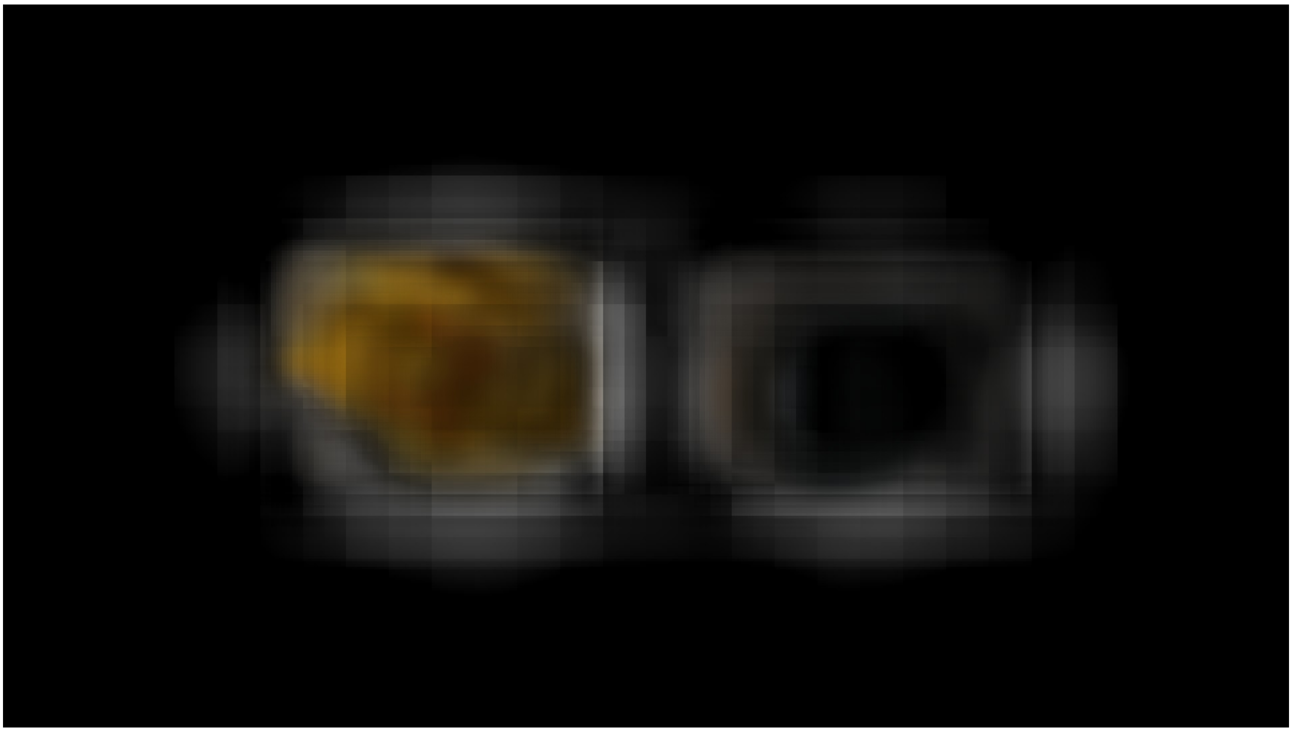

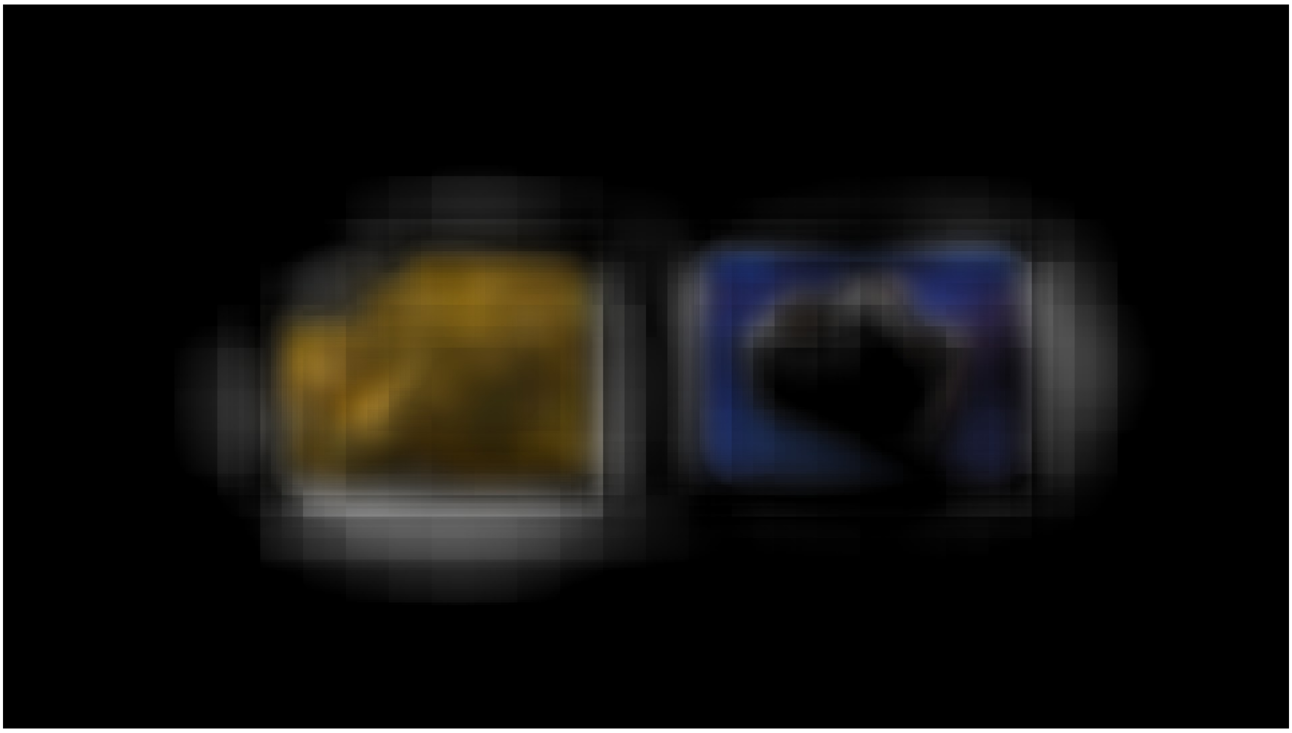

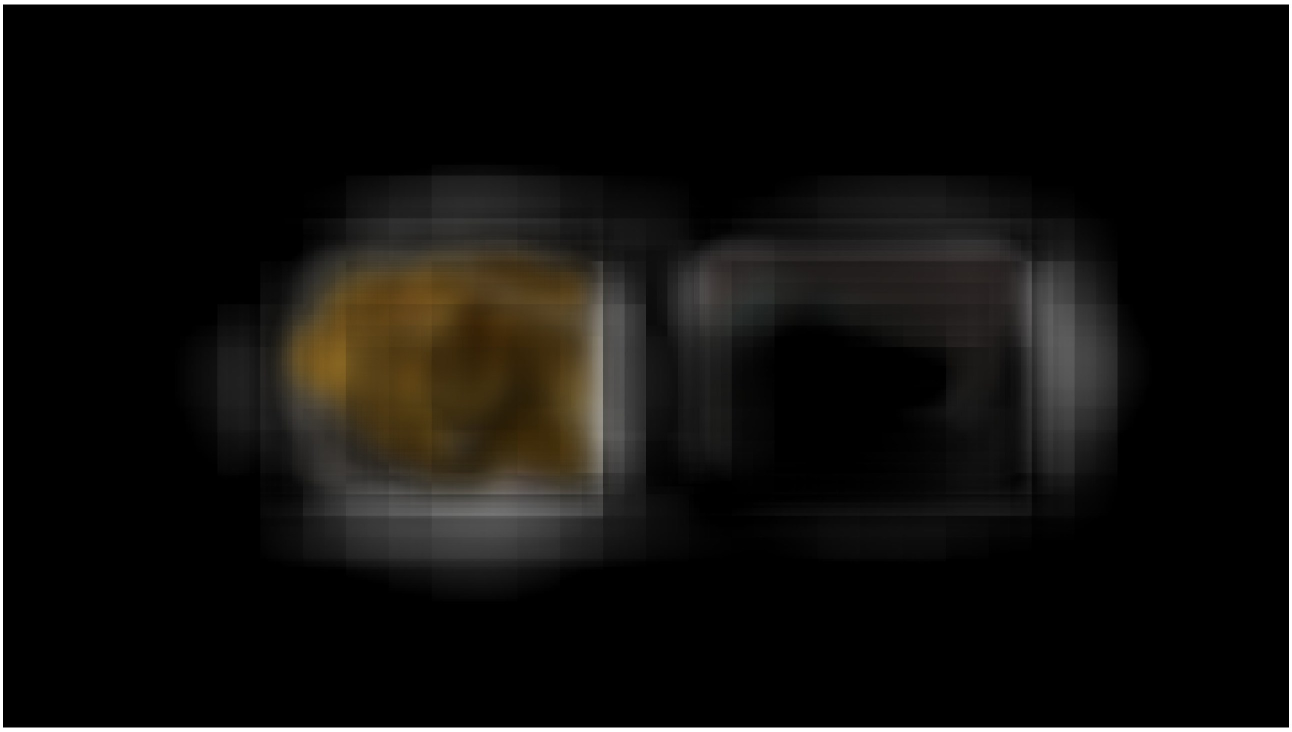

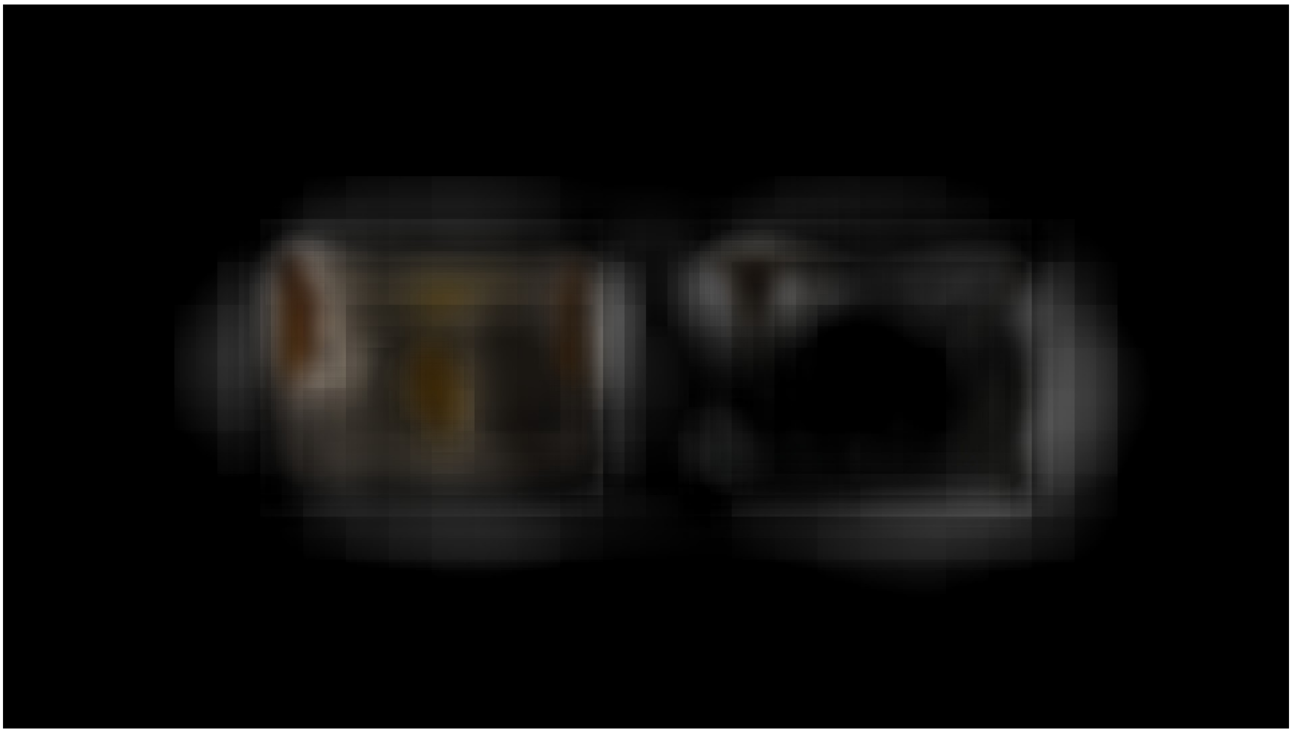

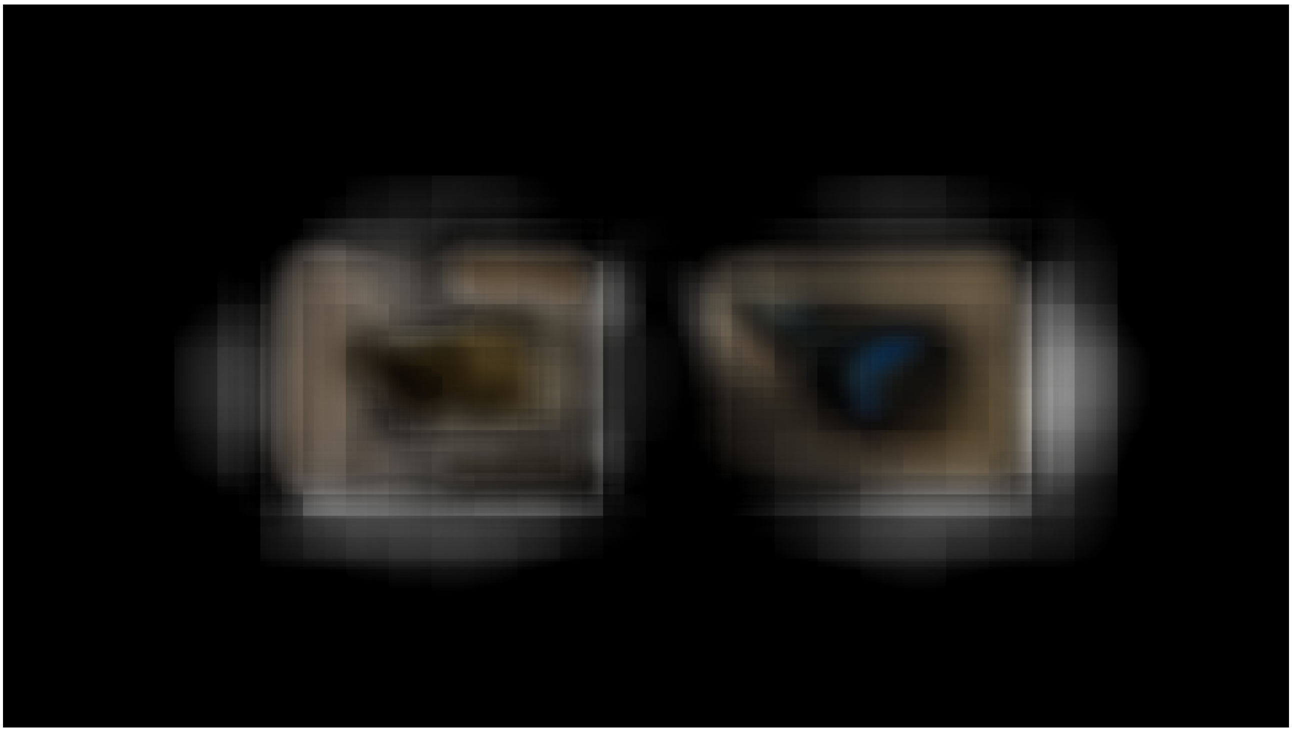

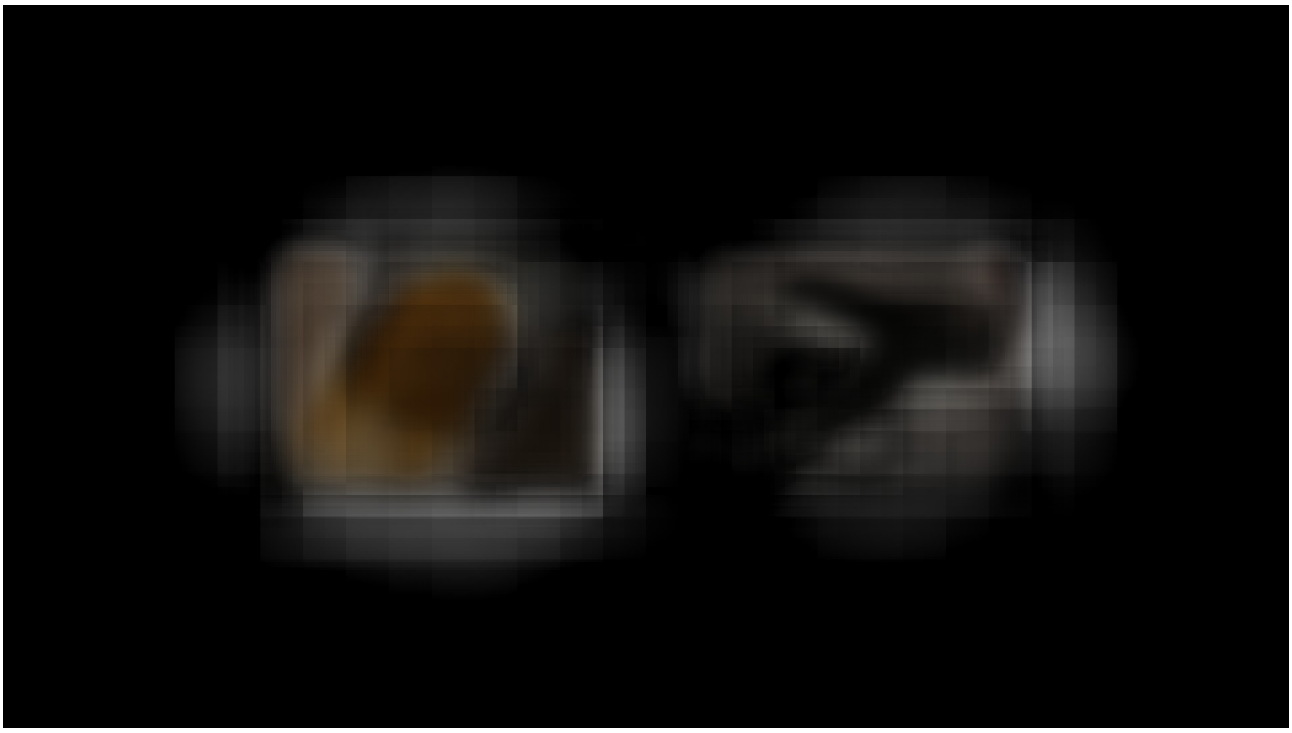

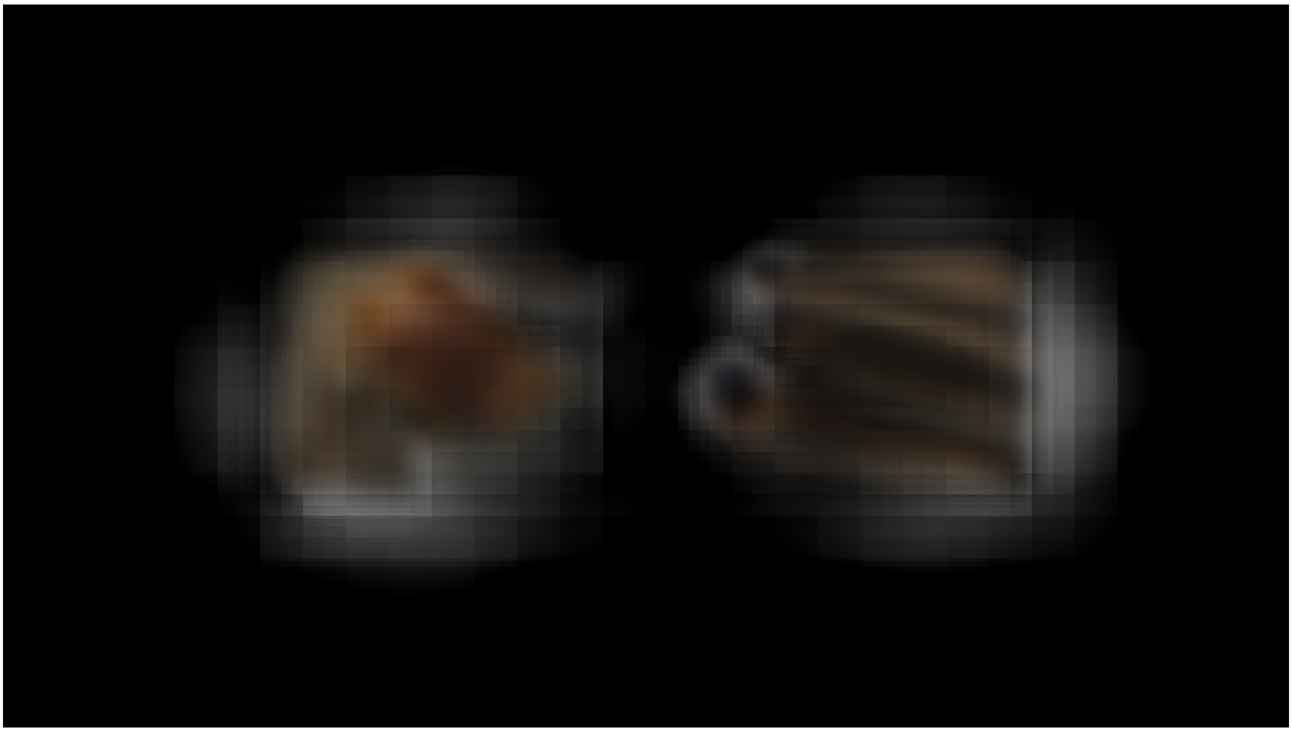

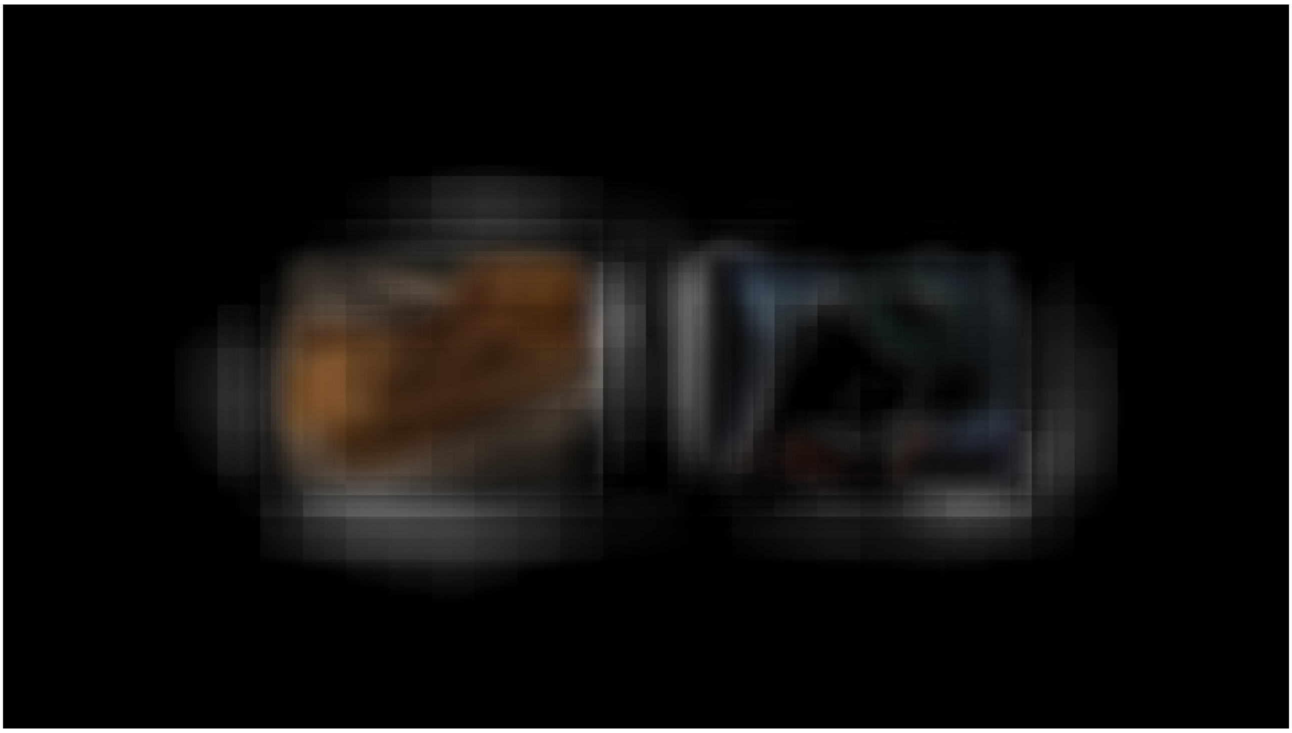

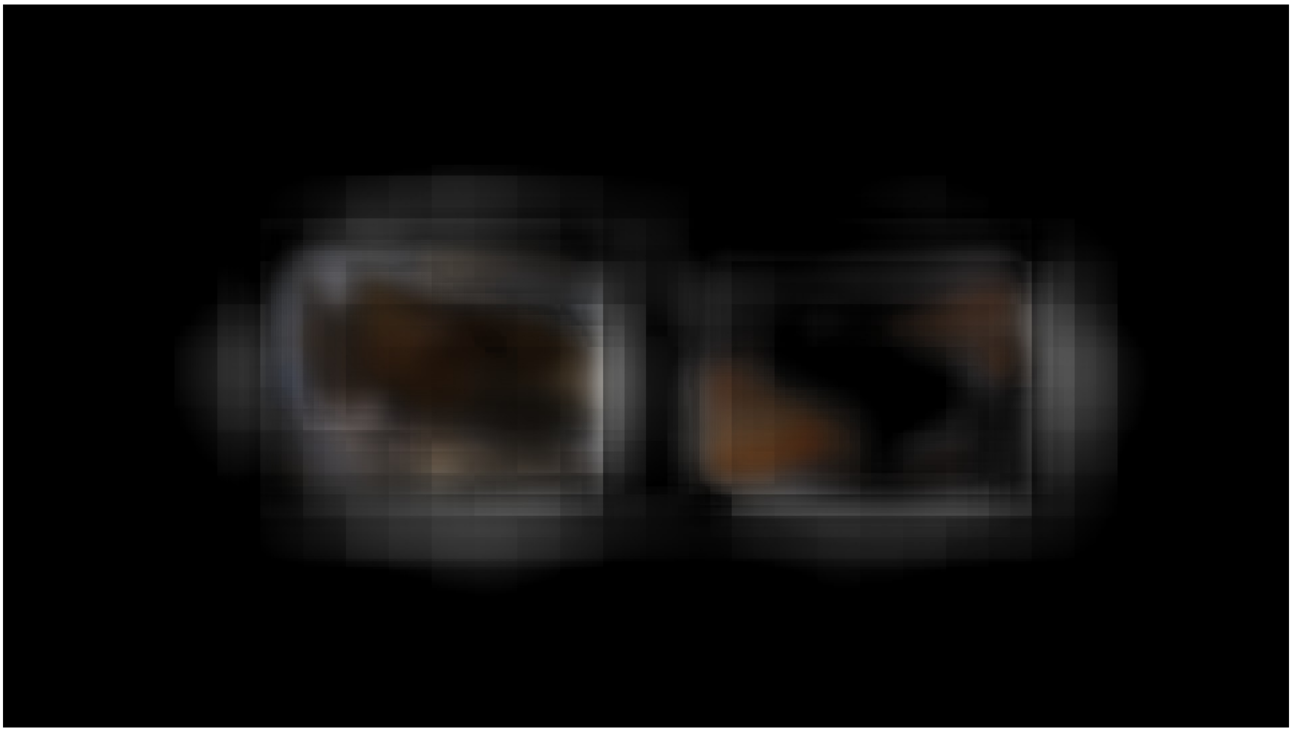

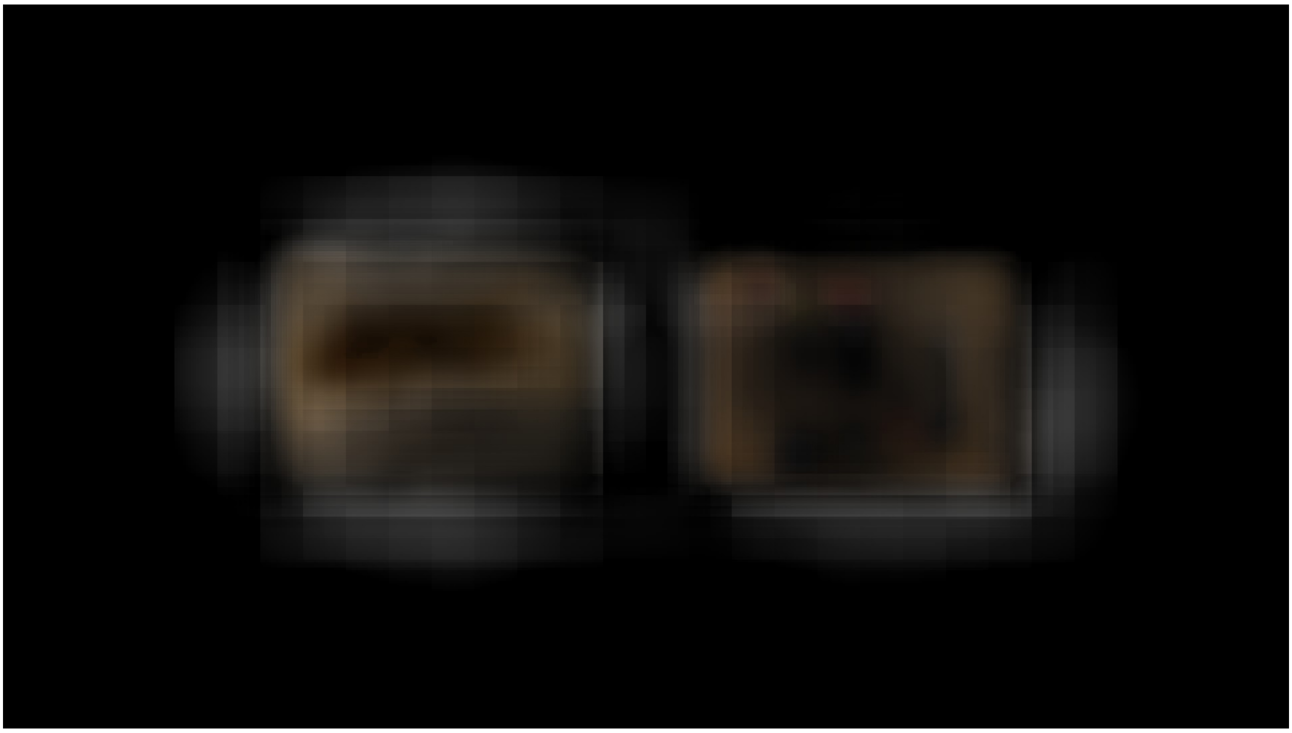

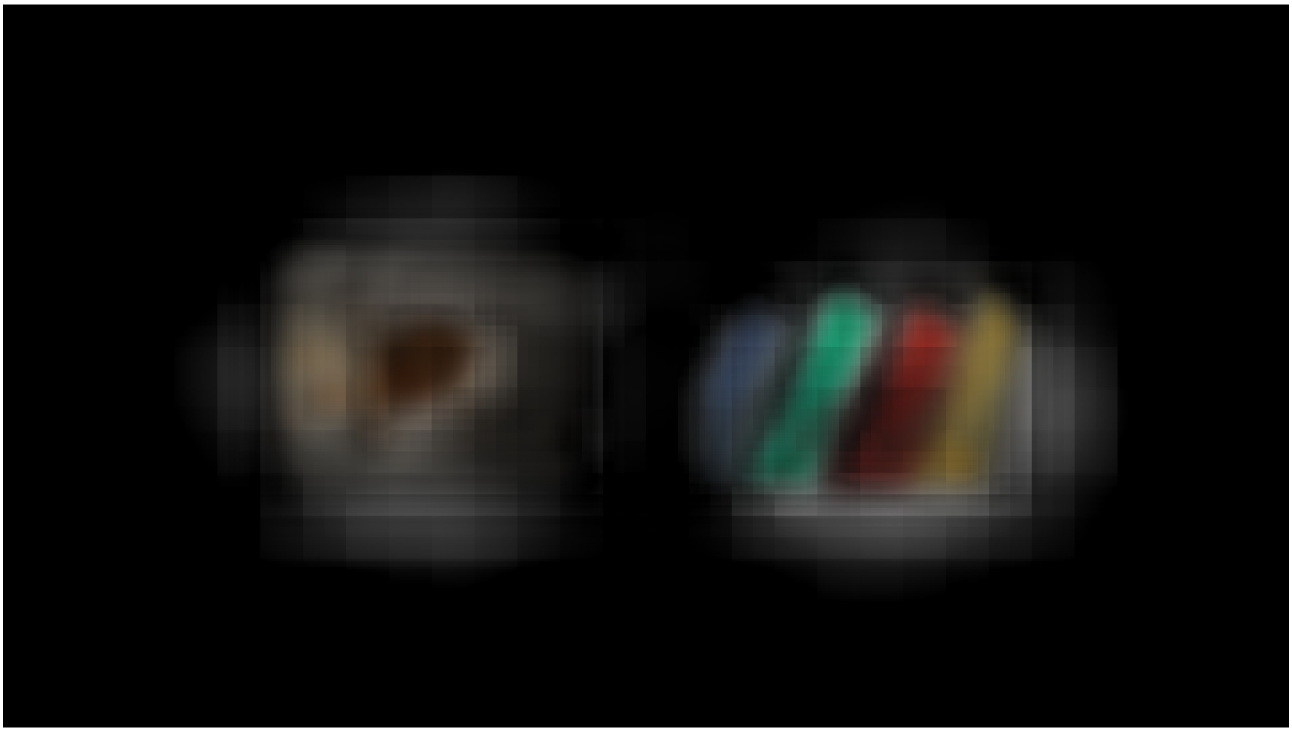

Supplement: Supplementary file 1 — Appendix S1: sjop70069‐sup‐0001‐AppendixS1.pdf. [file SJOP-67-787-s001.pdf]
